# SMOOTH-seq: single-cell genome sequencing of human cells on a third-generation sequencing platform

**DOI:** 10.1186/s13059-021-02406-y

**Published:** 2021-06-30

**Authors:** Xiaoying Fan, Cheng Yang, Wen Li, Xiuzhen Bai, Xin Zhou, Haoling Xie, Lu Wen, Fuchou Tang

**Affiliations:** 1 Bioland Laboratory (Guangzhou Regenerative Medicine and Health Guangdong Laboratory), Guangzhou, 510005 China; 2 Beijing Advanced Innovation Center for Genomics (ICG), School of Life Sciences, Department of General Surgery, Third Hospital, Peking University, Beijing, 100871 China; 3 Biomedical Pioneering Innovation Center, Ministry of Education Key Laboratory of Cell Proliferation and Differentiation, Beijing, 100871 China; 4 Peking-Tsinghua Center for Life Sciences, Academy for Advanced Interdisciplinary Studies, Peking University, Beijing, 100871 China

**Keywords:** Third-generation sequencing platform, Single-molecule sequencing, Single-cell genome sequencing, Structure variants, Extra-chromosomal circular DNAs

## Abstract

There is no effective way to detect structure variations (SVs) and extra-chromosomal circular DNAs (ecDNAs) at single-cell whole-genome level. Here, we develop a novel third-generation sequencing platform-based single-cell whole-genome sequencing (scWGS) method named SMOOTH-seq (single-molecule real-time sequencing of long fragments amplified through transposon insertion). We evaluate the method for detecting CNVs, SVs, and SNVs in human cancer cell lines and a colorectal cancer sample and show that SMOOTH-seq reliably and effectively detects SVs and ecDNAs in individual cells, but shows relatively limited accuracy in detection of CNVs and SNVs. SMOOTH-seq opens a new chapter in scWGS as it generates high fidelity reads of kilobases long.

**Supplementary Information:**

The online version contains supplementary material available at 10.1186/s13059-021-02406-y.

## Background

Single-cell whole-genome sequencing (scWGS) is a powerful tool to reveal cell to cell heterogeneity in biological samples and identify genomic changes such as copy number variations (CNVs) and point mutations [1–3]. Thus, the technology makes it possible to explore the cell lineages, especially the evolution of cells during tumorigenesis, and precisely digs out the lost heterogeneity information of bulk sequencing [3, 4]. In recent years, quite several single-cell genome amplification techniques have been developed, such as DOP-PCR [5], multiple displacement amplification (MDA) [6], multiple annealing and looping-based amplification cycles (MALBAC) [7], and Linear Amplification via Transposon Insertion (LIANTI) [8]. However, current scWGS methods are all based on next-generation sequencing platforms generating highly accurate but relatively short reads (several hundred base pairs), which are well-suited for calling copy number variations (CNVs), small indels, and single-nucleotide variations (SNVs), but not optimal for structural variations (SVs). SVs including deletions, insertions, duplications, and translocations in an individual cell have been rarely reported [9, 10], although they are one of the major sources of genetic variations in human somatic cells and can be driving forces for tumorigenesis and metastasis [11, 12]. Moreover, extra-chromosomal circular DNAs (ecDNAs) have recently been identified in human cells and supposed to play important roles in cancer development [13–15]. Especially, the long ecDNAs which are of hundreds to thousands of kilobase sizes can be highly amplified in cells and drive massive oncogene over-expression [16, 17]. These abnormal changes in the genomic structure may play critical roles for tumorigenesis and metastasis, yet there is no effective scWGS methods to systematically identify them.

In recent years, single-molecule real-time (SMRT) DNA sequencing technology has been used in investigating genome-wide SVs in the human genome [18]. The HiFi mode of PacBio platform generating long high-fidelity circular continuous consensus (CCS) reads of DNA templates achieved long read sequencing of high accuracy (99.8%) [19]. This makes it much easier and more reliable to map the reads directly spanning the breakpoints of SVs, since SVs often happen at repetitive element-enriched genomic regions, which are very challenging for mapping with hundred basepair-length short reads from NGS platform [19, 20]. However, the SMRT DNA sequencing usually needs microgram amount of DNAs as input, which introduces a great challenge in single-cell sequencing since an individual human cell only has several pictograms of genomic DNAs, which are millions of folds lower than needed.

To resolve the challenges in detecting SVs and ecDNAs in individual cells, we developed a single-cell genome sequencing method based on TGS platform and we named it as SMOOTH-seq (single-molecule real-time sequencing of long fragments amplified through transposon insertion). Tn5 transposition has been widely applied to construct shotgun fragment libraries for next-generation sequencing (NGS) [8, 21–23]. Different to previous designs, we embedded commercialized Tn5 transposase with one adaptor sequence instead of two different adaptor sequences. In this way, we could recover all of the original DNA fragments through transposition-PCR instead of only 50% of the genomic fragments with different adaptor sequences at their ends. Additionally, we optimized the reaction conditions and finally the proper reaction conditions we identified including concentration of the adaptor conjunct transpose, transposition buffer, and DNA polymerase enabled efficient long fragment capturing and amplification in an individual human cell. And these amplified long fragments are suitable for direct sequencing on the third-generation sequencing (TGS) platform, such as the SMRT DNA sequencing platform.

## Results

### The schematic of SMOOTH-seq analysis

**Fig. 1:**
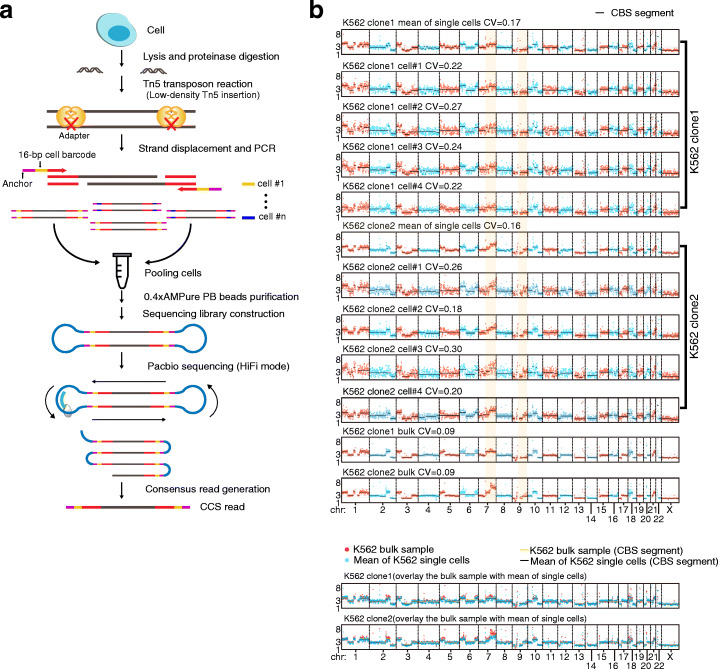
Schematic of SMOOTH-seq and CNV detection. **a** The schematic of SMOOTH-seq. After cell lysis and proteinase digestion, genomic DNA from a single cell is randomly fragmented by low-density Tn5 transposon insertion. Then, the produced fragments undergo strand displacement and amplification using 16bp-barcoded primers. Next, the amplified single cell gDNAs of different barcodes are pooled together and purified to prepare sequencing libraries. The libraries were sequenced on Pacbio Sequel II System using HiFi mode and the CCS reads are harvested for analyzing. **b** CNVs of single K562 cells showing in 1Mb windows (CV for each cell using bulk K562 copy number as the baseline). Digitized copy numbers across the genome are plotted in representative single K562 cells from clone 1 and clone 2 as well as the bulk samples of the two clones. The mean copy numbers are the averages of 44 cells from clone 1 and 47 cells from clone 2, respectively. The yellow shadow highlights the differences of CNVs on the long arm of chromosomes 7 and 9 between these two clones. At the bottom of pannel, the mean CNV values of K562 single cells to the CNV values of K562 bulk samples are plotted

To facilitate single-cell genomic DNA (gDNA) amplification with amplicons of proper length SMOOTH-seq (Fig. 1a), we firstly tested different transposition reaction buffers. Although the TBH buffer (offered with the commercialized Tn5 transposase) could achieve long fragments using 1ng genome DNA (gDNA) as input (Additional file 1: Figure S1a), this buffer did not work well for gDNA of picogram amount. An individual human diploid cell usually contains about 6 pg of genomic DNAs. The 5×TAPS_PEG8K performed robustly for a single-cell amount of gDNAs. We also found that the Tks DNA polymerase, which was reported to fit for long fragment amplification, could produce higher yield than the TAE (offered with the commercialized Tn5 transposase). When starting with 10pg of gDNA, we observed over amplification when the numbers of PCR cycles was 22, which leads to bias favoring shorter fragments (Additional file 1: Figure S1a). Reducing the concentration of Tn5 transposase could result in longer fragments, while it may also reduce the yield of amplicons (Additional file 1: Figure S1b). We finally used 0.2ng Tn5 transposase in a 10-μL transposition reaction for a single-cell sample and amplified its gDNA by 20 cycles of PCR using Tks enzyme (Additional file 1: Figure S1b). The amplicons after purification with 0.4 volume of AMPure PB beads were around 6kb long (Additional file 1: Figure S1c). We pooled amplified gDNA products of different cells with different barcodes together, and about 1μg amplicons was used for library construction of SMRT DNA sequencing. The amplified gDNA library was sequenced to generate CCS reads (Fig. 1a), which could reduce the TGS sequencing errors using subreads correction. The length of CCS was over 1kb and could reach as long as 43,693 bp, with the majority of the reads of about 6kb (Additional file 1: Figure S1d, e). The average polymerase read length was about 60kb for each library, indicating a mean of 10 passes (also known as subreads) for each CCS read, of which the read accuracy almost reaches Q30 (Phred quality score 30) [19]. These CCS reads were well mapped to the human genome, achieving 96% reads mapping ratio (Additional file 2: Table S1), which is much higher than that of single-cell NGS data [24].

### Detection of CNVs in individual K562 cells using SMOOTH-seq

We generated two clones of K562 cells (Additional file 1: Figure S2) and used SMOOTH-seq to analyze the genomes of individual cells from them (Additional file 1: Figure S3). Using pbmm2 v1.1.0 (https://github.com/PacificBiosciences/pbmm2) with --preset CCS, the CCS reads were mapped to human genome (hg38). The genome coverage increased with more CCS reads in single cells (Additional file 2: Table S1), ranging from 10.6 to 41.3% for the 91 K562 single cells (19.5% on average).

We first analyzed the CNVs of two clones of K562 cells. We directly calculated the reads ratios in every window at different bin sizes within each individual cell, and the CNV pattern for a single cell was relatively stable when calculating at 1 Mb windows. The coefficient of variation (CV), which is used to evaluate the noise of CNV calculation [8, 25] was 0.28 on average (Additional file 2: Table S1), and the baseline of copy number used to calculate CV is the mean value of all K562 single cells analyzed for each clone. Furthermore, we calculated the CV using bulk K562 cells of each clone as the baseline and got comparable CV values (Additional file 2: Table S1), we plotted the mean CNV values of K562 single cells to the CNV values of K562 bulk samples and can clearly see that they are highly consistent (Fig. 1b). In addition, we used circular binary segmentation algorithm to segment DNA copy number and plotted the heatmap for K562 bulk and single-cell samples (Additional file 1: Figure S4). Different individual cells within the same clone showed consistent CNV patterns. Clearly, we observed distinct CNV patterns between these two clones of K562 cells for chromosomes 7 and 9 (Fig. 1b and Additional file 1: S4), which could also be observed in the bulk NGS data (Fig. 1b), we further computed the Pearson correlation coefficients of segmented copy numbers between any two individual cells as well as between the single cells and the bulk samples (Additional file 1: Figure S5a and S5b, all *P*-value < 1×10^-5^). The single cells exhibited high correlations on CNVs, especially for those within each subclone. The correlations between single cells and the bulk samples are around 0.75, except for one cell showing relatively low correlation around 0.5. Overall, SMOOTH-seq showed acceptable performance on CNV analysis at the resolution of 1Mb.

### Detection of SVs using SMOOTH-seq

**Fig. 2:**
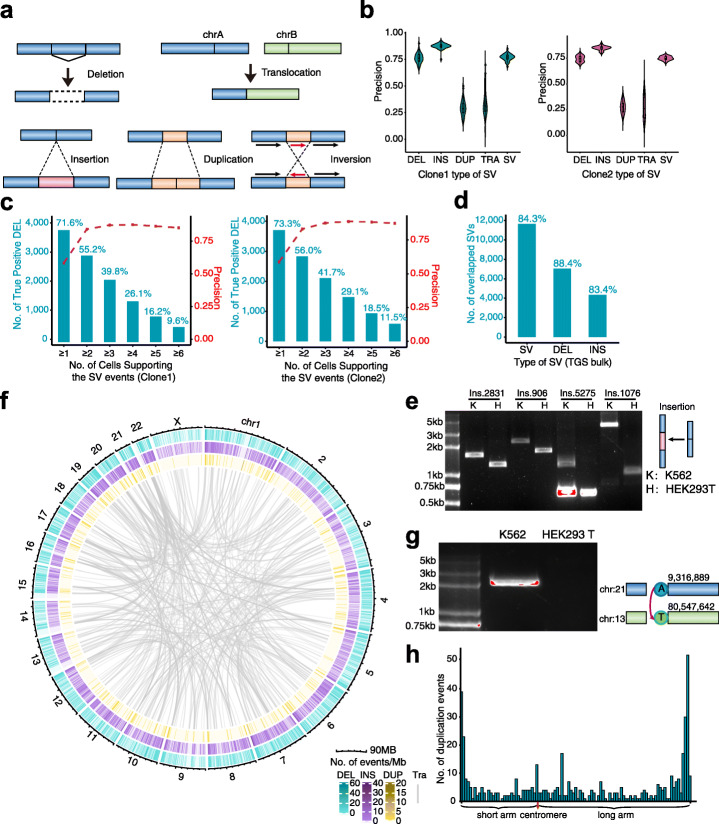
SV detection using SMOOTH-seq. **a** The diagram of insertions, deletions, duplications, and translocations and inversions. **b** Precision of SMOOTH-seq detecting SVs in clone 1 or clone 2 K562 cells. DEL, deletion. INS, insertion. DUP, duplication. TRA, translocation. **c** Precision of SMOOTH-seq detecting deletions and the percentage of true positive deletions with different numbers of supporting clone 1 or clone 2 K562 cells. **d** Overlapping of deletions and insertions detected by TGS bulk in clone 1 and clone 2. **e** PCR validation of 4 insertion events identified in K562 cells but not HEK293T cells by SMOOTH-seq. Ins, insertion. K, K562. H, HEK293T. ins.2831 represents insertions happened at chr17:57,539,630-57,539,631, ins.906 happened at chr14:31,993,906-31,993,907, ins.5275 happened at chr6:1,250,735-1,250,736, and ins.1076 happened at chr18:49,810,930-49,810,931. **f** A comprehensive overview of SVs detected by SMOOTH-seq in single K562 cells (shinyCircos was used for figure plotting [27]). **g** PCR validation of a translocation event in K562 cells but not HEK293T cells. The diagram on the right indicates the detailed positions and directions of the translocation. **h** Distribution of duplication events on the chromosomes. The chromosomes are separated into 100 windows from the centromere to the telomere. The number of duplication events are calculated in each window. The  uniform distribution test was conducted and the *P-*value was less than 1×10^-3^

SVs in the genome could usually be classified into deletions, insertions, translocations, duplications, and inversions [26] (Fig. 2a). Here, we used pbsv (https://github.com/PacificBiosciences/pbsv) to call SVs in single K562 cells of the two clones (Additional file 1: Figure S3). The TGS-based genome sequencing method using bulk samples is now well-established technology [20, 28]. It has been systematically evaluated and is approved to be able to accurately detect genomic SVs (>50bp) owing to the long read length, especially for Pacbio platform [19]. So we sequenced bulk genome of the two K562 clones on the Pacbio as gold standard to evaluate our SMOOTH-seq method for SV detection (Additional file 2: Table S1, Additional file 1: Figure S1e). We identified the SVs in the bulk using pbsv (Additional file 1: Figure S6a). We further compared the SVs obtained in single cells to those in bulk and filtered indels less than 100bp long, which showed frequent sequencing errors in TGS platform. The SV detection precision in each single cell of both clones was high (76.9%, 75.2% on average) (Fig. 2b). For all types of SVs, insertion turned to be the most accurate mutations (87%, 84% on average) detected in single cells, and deletions ranked the second. Duplication, which is quite difficult to be identified using short-read sequencing technology, also appeared with the least precision rate (no more than 60%) in single-cell data of SMOOTH-seq (Fig. 2b). As expected, precision of the SVs (mainly deletions and insertions) increased with growing number of supporting cells in both clones, especially from one-cell-supported to two-cell-supported SVs (Fig. 2c, Additional file 1: S6b). Accordingly, the false positive rate (FPR) and harmonic mean (see methods) showed a clear downtrend from at least one-cell-supported to multicell-supported for both deletions and insertions (Additional file 1: Figure S6c). While the false negative rate showed uptrend accordingly. Compared to the FPR of at least one-cell-supported deletions (48.6%), the FPR of at least two-cell-supported deletions drops sharply to 17.8%, indicating the effectiveness of cell-sharing strategy to increase the precision of SV detection. To further determine the performance of SV detection, we used two other metrics including recall and F1-score for analysis (Additional file 1: Figure S6d). The downtrend of these metrics with multicell supporting showed similar results. Therefore, we chose the SV events supported by at least two cells for all subsequent analyses. Meanwhile, the number of true positive SVs decreased by asking more supporting cells (Fig. 2b, Additional file 1: S6b). In both clones, about 55.2% (56.0%) of the deletions and 42.8% (43.0%) of the insertions in bulk could be identified with two supported cells. When there were more cells by merging two clones, the detection efficiency of two supported cells could improve to 73.9% and 60.6% for deletions and insertions, respectively (Additional file 1: Figure S6e).

To see if sequencing a single-cell deeper and cover more percentage of its genome will provide additional advantage, we picked four K562 single cells with the highest genome coverage (35.7–41.2%) in our dataset to perform further analysis. As shown in Additional file 1: Figure S7, the detection of two-read-supported SV events in a single cell is much more accurate than that of one-read-supported SV events. So further increasing the sequencing depth for a single cell would be helpful to further improve the accuracy of calling SVs if the high sequencing cost is not considered.

We then compared the similarity of these SVs between the two clones based on bulk analysis. Both clones shared almost the same length distribution for different SV types (Additional file 1: Figure S8a, b). Further comparison showed that more than 80% SVs were shared between these two clones, indicating high similarity of genome states between them (Fig. 2d). To recover as many SVs from single-cell data as possible, we merged single cells of the two clones for further analysis.

From the total 91 single K562 cells, we identified thousands of SV events by requiring two supported cells. Globally, with longer reads in bulk we could identify more large-scale SVs (Additional file 1: Figure S8a-c). More than half of the deletion and insertion events (77.9% and 77.3%, respectively) happened within 500bp, and the vast majority (87.2% and 90.8%, respectively) were less than 1kb (Additional file 1: Figure S8c), while the ratios of SV events less than 1kb in bulk samples were 83.1% and 82.1% for deletions and insertions, respectively. Still, the longest deletion event detected was 89,252bp, which was supported by 8 individual cells, and all deletions detected by SMOOTH-seq showed high consistency (85% overlap) with bulk TGS data (Fig. 2c, Additional file 1: Figure S8d), verifying the accuracy of SMOOTH-seq in detecting deletion events. The NGS bulk also showed comparable ability to detect deletion events (Additional file 1: Figure S8d). For the insertion events, the longest detected in bulk TGS data was 9833 bp, while the single cells could detect as long as 7780 bp in this dataset. The single-cell results were also quite consistent with those found in bulk TGS data (94% overlap) (Additional file 1: Figure S8e). As expected, with much longer reads we found many more insertion events using SMOOTH-seq than previous bulk NGS-based studies [29] (Additional file 1: Figure S8e), reflecting its outstanding performance than short-read NGS-based sequencing methods. This turned out the same situation for inversions that SMOOTH-seq detected more events than the NGS bulk sequencing did, and the length of inversion events detected could reach megabases (Additional file 1: Figure S8f) [29]. As a complement, we picked 8 insertion events from the K562 SMOOTH-seq analysis for PCR validation. Four insertions were validated with the exact insertion sizes (299bp, 455bp, 598bp, 2631bp, respectively) in K562 gDNA (Fig. 2e). For the left 4 insertion events, PCR results showed that both K562 gDNA and a control gDNA sample (HEK293T) have these insertion events in one of their alleles (data not shown).

As a chronic myelogenous leukemia (CML) cell line, K562 has been reported to carry several translocations which leads to fusion genes. For example, translocation of chr9 with chr22 generates *BCR-ABL1* fusion gene [30]. We further checked the translocation events in individual K562 cells by SMOOTH-seq. A total of 521 translocation events were identified and all of them happened interchromosomally (Fig. 2f, Additional file 1: Figure S6a). We detected *BCR-ABL1* fusion events in 51 out of 91 K562 cells, and in the left cells, we only captured the wild type alleles. Except for reads supporting fusion of *BCR* and *ABL1*, we also obtained reads containing *NUP214-XKR3* fusion gene, which has been a known translocation event in the K562 cell line [31, 32] (Additional file 1: Figure S8g). Additionally, we validated the chr13:80,547,642-chr21:9,316,889 translocation in K562 cells by PCR using HEK293T cells as control (Fig. 2g). These results indicate that SMOOTH-seq is a reliable and robust scWGS method to identify translocation events.

Duplications in the genome are composed of repeat units that linked to each other in head-to-tail manner for at least twice (Fig. 2a). By SMOOTH-seq, we identified 485 duplication events in the K562 cells (Additional file 1: Figure S6a). The length of the repeat units varied from 100 to 5911bp (Additional file 1: Figure S8c), and we noticed that most of the duplication events occurred in the genomic regions near the telomeres (Fig. 2f). We further separated chromosome into 100 windows from the centromeres to the telomeres and calculated the number of duplication events in each window (Fig. 2h). Indeed, the duplication events happened more in the windows near the telomeres (*P*-value<1×10^-3^, Fig. 2h), indicating that genomic regions near the telomeres have more chances for genomic duplication events in K562 cells.

### Detection of ecDNA in K562 by SMOOTH-seq

**Fig. 3:**
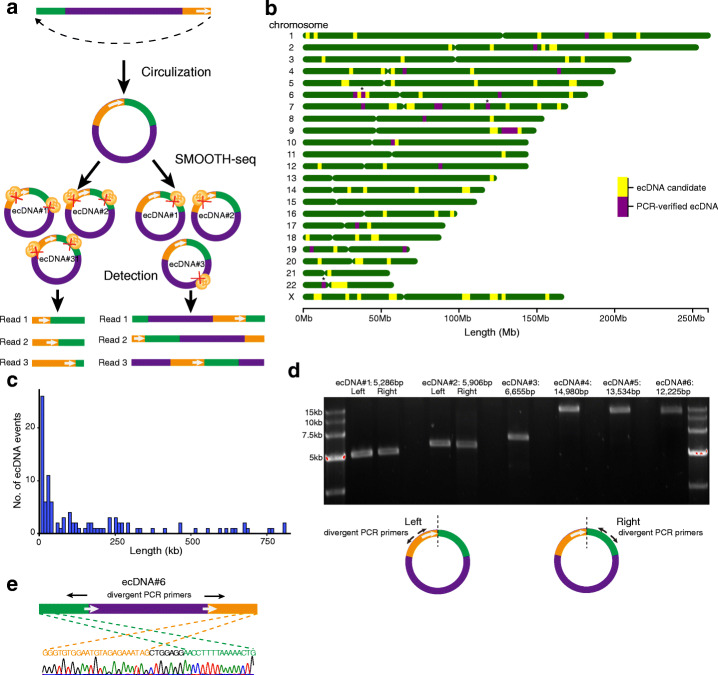
ecDNA detection in K562 cells. **a** Diagram showing the detection of ecDNAs by SMOOTH-seq. The green part and orange part show the flanking regions of the circulation sites. The Tn5 transposases bind on an ecDNA molecule, generates linear fragments spanning the circulation site. When only one copy of Tn5 transposases inserts into an ecDNA, it will produce linearized read sequence of the full ecDNA length. **b** The location distribution of candidate ecDNAs (yellow) and PCR verified ecDNAs (purple) on the chromosomes (R package chromoMap v0.2 was used for figure plotting). The stars indicate regional overlap of both unvalidated and validated ecDNAs. **c** The length distribution of ecDNAs (125 in total) detected in K562 cells. **d** Validation of six ecDNAs in K562 cells by full-length PCR using divergent primers as indicated in the bottom. ecDNA#1 represents chr8: 70,427,292-70,432,578, ecDNA#2 represents chr6:117,278,014-117,283,920, ecDNA#3 represents chr19:4,218,299-4,224,954, ecDNA#4 represents chr19:58,198,975-58,213,955, ecDNA#5 represents chr2:140,078,412-140,091,946, ecDNA#6 represents chr6:35,786,783-35,799,008. **e** Sanger sequencing result of the circularization site of ecDNA#6 in panel **d**

EcDNAs are common during carcinogenesis and oncogenes can be amplified in ecDNAs [15]. They have variable sizes ranging from kilobases to megabases. To figure out the ability of SMOOTH-seq to detect ecDNAs, we developed a pipeline to identify ecDNAs in K562 cells (Fig. 3a) (see methods). The long read length of SMOOTH-seq made it possible to capture the full-length ecDNAs less than 10kb in a single sequencing read. When only one copy of Tn5 transposase binding on an ecDNA molecule, the whole circular DNA molecule could be amplified into one linear fragment that cover its full-length sequence (Fig. 3a). In this way, we could detect different reads of exactly the same length for different copies of the same ecDNA, which is a feature that does not exist in tandem repeats (Fig. 3a). The mitochondrion DNAs, which are the most abundant circular DNA in cells were analyzed at first to validate our method. We obtained quite abundant mitochondrion DNA reads in the K562 cells, and the longest read was 16,550bp, almost reached the full length (16,569bp), indicating high sensitivity of SMOOTH-seq in detecting circular DNAs (Additional file 1: Figure S9a). In addition, we identified a total of 125 candidate ecDNAs supported by reads from at least two individual K562 cells (Fig. 3b, Additional file 3: Table S2). These ecDNAs mainly ranged from 5kb to 1Mb (Fig. 3c), 29.6% of which contained genes inside. A total of 417 genes were identified within ecDNA regions, and the gene ontology of these genes enriched in the biological terms of signaling pathways regarding immune processes and cell divisions (Additional file 1: Figure S9b) [16]. 90% selected ecDNA candidates (18 out of 20) could be verified by PCR coupled with Sanger sequencing (Fig. 3e, Additional file 1: S10 and Additional file 4: Table S3). For 8 ecDNA candidates less than 15kb, we also amplified the full-length sequences using divergent primer pairs (Fig. 3d). Six out of the 8 ecDNA candidates were successfully amplified with expected full-length sizes, and the circularization sites were further validated by PCR coupled with Sanger sequencing (Fig. 3d, e). Taken together, SMOOTH-seq can accurately detect ecDNAs in individual cells.

### Detection of SNVs in K562 cells

**Fig. 4:**
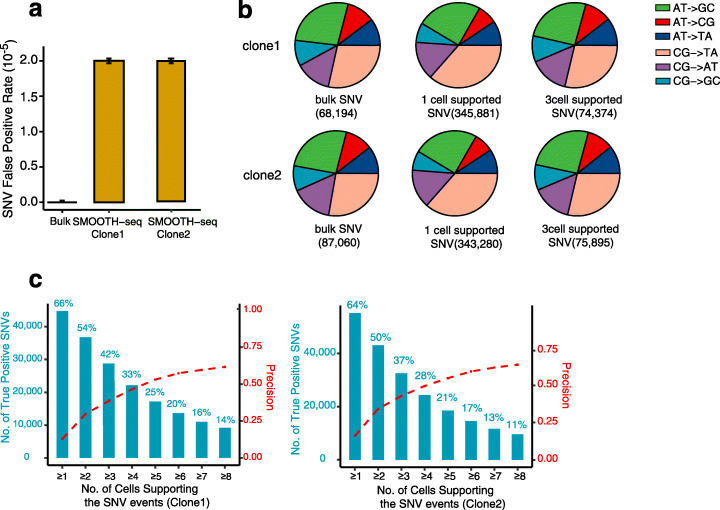
SNV detection in K562 cells. **a** False positive rate (FPR) of SMOOTH-seq in detecting SNVs in single K562 cells. The error bar generates according to the values in 95 single cells. The bulk SNVs are regarded as the standard reference with the FPR as 0. **b** Spectra of SNVs in bulk and SMOOTH-seq of K562 genome. SMOOTH-seq exhibit slightly biased CG-to-TA false positives in one K562 cells. **c** Precision of SMOOTH-seq detecting SNVs and the percentage of true positive SNVs with different numbers of supporting clone 1 or clone 2 K562 cells

In terms of SNV detection, we first evaluated the accuracy of calling SNVs in single cells using bulk NGS sequencing data as controls. GATK was used to call SNVs in both single K562 single cells (where ploidy was set to one, as the coverage depth of CCS reads is less than 1× and was not adequate to differentiate heterozygous and homozygous SNVs, see the “Methods” section) and bulk samples [33]. We removed single-nucleotide polymorphism (SNP) sites when searching the candidate mutations. It is worth mentioning that we were detecting “germline-like” mutations of the K562 cells, since it is a cultured cell line which was subcloned already. A total of 68,194 and 87,060 SNVs were called in the bulk NGS samples of the two K562 clones, of which an individual cell covered 7.5% and 6.7% on average. We followed the previous published workflow to calculate the SNV false positive rate in each single cell by dividing the number of the false positive SNVs by the total covered bases in a single cell [7]. As the average number of candidates SNVs called in a single cell was 16,488, the false-positive rate for each single-cell SNV calling was 2.0 × 10^-5^ on average (Fig. 4a, see the “Methods” section). We further characterized the spectra of false positives and found that SMOOTH-seq showed preference for CG-to-TA false positives when asking the SNV supported by any one individual cell (Fig. 4b). This kind of false positives has been widely reported in many scWGS studies and SMOOTH-seq showed better performance than previous scWGS methods [6, 8, 34]. Moreover, the preference of candidate SNVs could be corrected when we asked the SNVs supported by more individual cells (Fig. 4b and Additional file 1: S11a).

We then checked the efficiency and precision of detecting true SNVs (called in bulk) in single cells using SMOOTH-seq. Within both K562 clones, the SNV detection efficiency (number of true positive SNV/bulk NGS detected SNV) decreased dramatically along with the increasing of the supported cell number due to relatively low genome coverage of each cell. Meanwhile, the SNV detection precision (number of true positive SNV/(true positive SNV + false positive SNV)) increased (Fig. 4c), and the precision clearly increased when we use more supported cells to correct the SNV results (Additional file 1: Figure S11b). By asking the SNVs supported by 8 individual cells, the detection efficiency was reduced to no more than 14% whereas the precision reached over 60% in both clones.

### SV detection in colon cancer cells using SMOOTH-seq

**Fig. 5:**
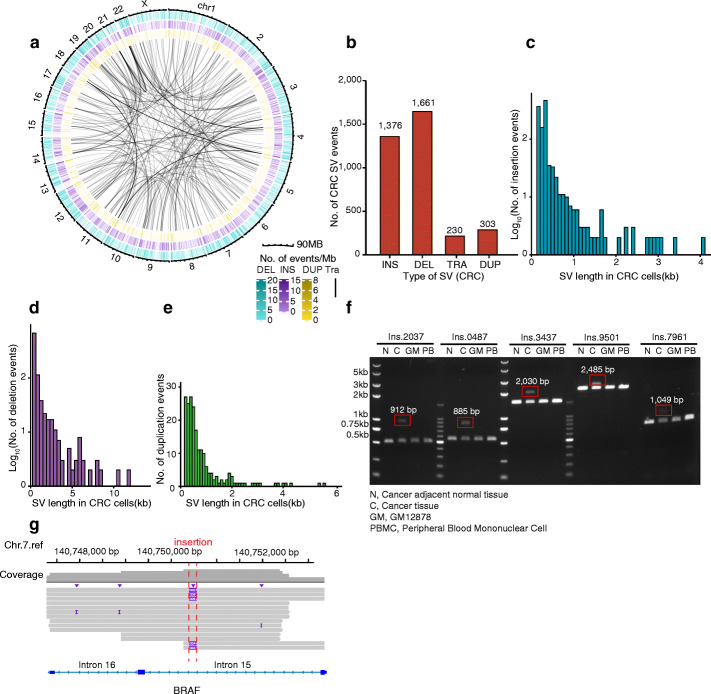
SV detection in colorectal carcinoma (CRC) cells using SMOOTH-seq. **a** A comprehensive overview of SVs detected by SMOOTH-seq in single CRC cells. **b** The number of insertions, deletions, translocations, and duplications events in a patient’s colorectal carcinoma sample. **c**, **d**, **e** Histograms of length distributions for insertion, deletion, and duplication events detected by SMOOTH-seq in CRC cells. **f** PCR validation of insertions events in cancer tissue but not existing in normal tissue, GM 12878, or peripheral blood mononuclear cell. N, cancer adjacent normal tissue. C, cancer tissue. GM, GM 12878. PB, peripheral blood mononuclear cell. **g** An example of insertions in cancer tissue whose insertion site located at the intron of BRAF gene. **h** The location distributions of ecDNA in cancer tissue on chromosomes

To further check the ability of SMOOTH-seq detecting SVs in in vivo cells, we collected single-tumor cells from a colorectal cancer patient and performed SMOOTH-seq on 96 cells. By requiring at least 5% genome coverage, 44 cells were left for SV investigation (Additional file 2: Table S1). These cells showed large scaled CNVs (Additional file 1: Figure S12a), representing a tumor cell character. By asking the SVs supported by at least 2 colorectal cancer cells (CRCs), we identified 4089 insertion events, 3852 deletion events, 341 translocation events, and 312 duplications. The same as K562 cell lines, the translocations happened almost inter-chromosomes and the duplications enriched near the telomeres (Additional file 1: Figure S12b). 95.1% of the insertion events were less than 1kb and 97.5% of the deletion events were happened within 5kb. The long reads enabled us to identify duplications longer than 5kb (Fig. 5c–e). We extracted the CRC-specific SV mutations by removing those overlapped with the SVs identified in K562 cells. 3570 SV events (1376 insertions, 1661 deletions, 230 translocations, and 303 duplications) were retained as CRC-specific. SMOOTH-seq showed excellent performance on detecting insertion events, 5 out of 8 picked candidates were validated by PCR (Fig. 5f), including a 330-bp insertion in an intron of BRAF (Fig. 5g). The majority of the CRC-specific SVs happened in the intergenic region, and 1547 caused changes in the gene regions (Additional file 5: Table S4). To our surprise, 5019 of all the SVs called in CRCs were the same as those found in K562 TGS data. We further checked these SVs using PCR in a couple of gDNA samples, including the corresponding tumor tissue, tumor adjacent normal tissue, an B cell line GM12878, and the peripheral blood mononuclear cells from another person. We found the PCR results were the same in all the gDNA samples, different from the suspected size in the current genome reference, but including the size of each SVs (Additional file 1: Figure S12c). We suspected that the current human genome annotation was still imperfect and the third-generation sequencing would be helpful in reconstructing a more complete genome reference.

## Discussion

We presented a novel method SMOOTH-seq for single-cell genome analysis based on SMRT sequencing technology, which enabled accurate SV detection by taking advantages of long high-fidelity reads. Like other scWGS methods, we can obtain the CNV and SNV information from an individual cell. Ideally, it is better to sequence individual cells to enough depth to make comprehensive evaluation of a method. However, due to the high price of SMRT sequencing comparing to NGS at current stage, it is unrealistic to sequence a cell by SMOOTH-seq to the adequate depth as NGS does. In this study, we pooled about 16 cells in a sequencing library, which could generate around 400Gb data from the Pacbio Sequel II platform per run. While these are only about 15Gb CCS data, corresponding to 1Gb for each individual cell. Even so, the total cost for one individual cell was ~$260, which made it very expensive for large number of cells.

Previous applications used Tn5 transposition containing two adaptors with different sequences to prevent fragment self-looping. However, only half of the DNA fragments will be tagged by different adaptors on both ends, leading to 50% loss of the original DNA fragments during sequencing. In SMOOTH-seq, we used just one Tn5 adaptor sequence to avoid original DNA fragments losing. Meanwhile, the long fragments generated by low-concentrated Tn5 transposase reduces the chance of self-looping. It is very unlikely that the same genomic sites on different alleles are cut identically by the transposases by chance; thus, we can easily remove duplicated reads from overamplification of the same gDNA fragment, as the previous transposition-based WGS method did [8]. However, as an expense, we got limited genomic coverage (an average of 19% of the genome) and sequencing depth (an average of 0.4× depth) of each individual cell. For the same reason, the sensitivity and accuracy of calling CNVs and SNVs should also be improved with more sequencing data for each cell. This would not be a problem when the cost of TGS sequencing decreases to the same level as that of the NGS in the future.

There is no doubt that SMOOTH-seq showed well performance on SV detection, especially for insertion, translocation, and duplication events in both cell lines and in vivo cancer cells. We can directly capture the complete variant structures instead of deducing them by reads assembly. This has additional advantages when there are repeats inside the variant structures. We infer that that is why we observed many duplication events flanking the telomere of the chromosome [34], while other NGS based methods are difficult to detect such SV events. PCR chimera artifacts [24, 35, 36] could not be distinguished from SVs such as translocation events, while these random events could be excluded by requiring the SVs identified in multiple individual cell samples.

We are excited that SMOOTH-seq offers a way to investigate ecDNAs at single-cell level, a new type of SVs identified recently. A single transposition event on a single-ecDNA molecule made it possible to capture the full-length ecDNA. And when the ecDNA is relatively short (generally no more than 10kb), a single-sequencing read could recover its full-length sequence. However, longer candidate ecDNAs can only be detected by the circularization sites, which are difficult to be distinguished from the large duplication events. And currently, no effective way can distinguish ecDNAs and duplication events. In this analysis, by removing the candidates overlapped with duplication events, we eliminated the interference from duplication events to a certain extent. It is worth mentioned that we supposed and captured the ecDNAs with the simplest sequence structures, ecDNAs with more complex structures could be probably found when there are more appropriate analysis pipelines for long sequencing reads. In all, SMOOTH-seq made a breakthrough on scWGS analysis, which especially facilitates for single cell SV and ecDNA detection.

## Conclusions

SMOOTH-seq is a breakthrough of scWGS by utilizing the third-generation sequencing platform to obtain much longer genomic reads. This method reliably and effectively detected SVs and ecDNAs in individual cells, but detected CNVs and SNVs with less accuracy. We believe the method has wide application potential in single-cell genomics field.

## Methods

### The aim of study

There is no effective way to detect structure variations (SVs) and extra-chromosomal circular DNAs (ecDNAs) at single-cell whole-genome level. So we developed SMOOTH-seq for single-cell genome analysis based on SMRT sequencing technology, which enabled accurate SV detection by taking advantages of long high-fidelity reads.

### Culture of K562 and human HEK293T cells

K562 cells were maintained in RPMI1640 with 10% FBS, 1× l-glutamine and 1× Pen/Strep. HEK293T cells were maintained in Dulbecco’s modified Eagle’s medium (DMEM)/high glucose with 10% FBS, 1× l-glutamine and 1× Pen/Strep. All cell culture reagents were purchased from Gibco.

### Bulk DNA extraction and whole genome sequencing library construction

Genomic DNA (gDNA) was extracted using the QIAGEN DNeasy Blood and Tissue Kit (QIAGEN) following the mammal’s instructions. The extracted gDNA was quantified using the Qubit dsDNA HS Assay Kit (Invitrogen), and approximately, 500 ng gDNA was sheared into around 300 bp fragments using Covaris S220, subsequently for library construction with KAPA Hyper Prep Kit. The final cycle for library amplification was 4 to reduce PCR bias. The samples were sequenced on illumina HiSeq-4000 with 150 bp pair-end reads.

### SMOOTH-seq library preparation of single cells

Individual cells were collected with a microcapillary connected to a mouth pipette and washed by transferring them into droplets of 1 mg/mL phosphate-buffered saline-bovine serum albumin for three times before lysis. The 2.5-μL lysis reaction consists of 0.25-μL 100mM Tris-EDTA (1M Tris + 0.1M EDTA), 0.125μL Qiagen protease, 0.075μL 10% triton X-100, 0.05μL 1M KCL, and 2μL H_2_O. The cell lysis was carried out at 50°C for 3 h to digest the proteins binding on the gDNA and then 70°C for 30 min to inactivate the protease. After that, a 7.5-μL tagmentation mixture including 2 μL 5×TAPS_PEG8K (50 mM TAPS-NaOH (or KOH), pH 8.3 (RT), 25 mM MgCl_2_, 40% PEG8K), and 1μL 0.2ng/μL adaptor conjuncted Tn5 enzyme (Vazyme, Cat. S601-01) was added into each cell lysate. The tagmentation reaction was carried out at 55°C for 10min, followed by adding 2.5-μL 0.2% SDS and standing at room temperature for 5min to stop tagmentation, releasing the fragmented gDNA. Then, strand displacement of the Tn5 adaptors and amplification of the fragmented gDNA was carried out using 0.025U/μL Tks Gflex DNA Polymerase (TAKARA, Cat. R060B), 560nM I5 PCR primer which containing 16 bp cell barcode (5′ AATGATACGGCGACCACCGAGATCTNNNNNNNNNNNNNNNNTCGTCGGCAGCGTC3′). The PCR program was 72°C 3min, 98°C 1 min, and then 20 cycles of 98°C for 15s, 60°C for 30s, and 68°C for 5min. After that, gDNA amplicons using different barcode primers were pooled together and purified with 0.4 volume of Ampure PB beads (Pacific Biosciences Ref. No. 100-265-900) for twice. These purified amplicons were quantified using Qubit, and about 1μg amplified products was used to construct libraries for Pacbio sequencing using SMRTbell Template Prep Kit v.1.0-SPv3 (Pacific Biosciences Ref. No. 100-991-900).

### Validation of structure variations in K562 and HEK293T cells

Insertions and translocations in K562 called from SMOOTH-seq were selected for PCR validation. PCR primers were designed such that the amplicons span the breakpoints and produce PCR products. Then, the HEK293T cell gDNA amplified with the same primers were used as control. Amplification was done by Tks Gflex DNA Polymerase. All PCR amplicons were run on 1% agarose gels.

EcDNAs in K562 were picked for verification. For circDNAs <15kb, we designed two pairs of outward PCR primers at left/right positions of circularization site to amplify the whole ecDNA sequence. For ecDNAs >15kb, we designed a pair of inward PCR primers to amplify sequences flanking the circularization site. Amplification was done by 2×Taq PCR Mastermix (TIANGEN,Cat.KT201). The PCR products of expected length over 5kb were then run on 0.8% agarose gel and those less than 1kb were run on 1.5% agarose gel. The primers used for validation were summarized in Additional file 5: Table S4. The strands of inward PCR products were cut out directly for Sanger sequencing to confirm circularization sequences.

### Patient sample

Sample was collected from a patient pathologically diagnosed with microsatellite-stable CRC. Matched adjacent normal tissues and cancer tissues were obtained from the same patient. Informed consent was acquired from this patient. Sample and experimental steps in this study were approved by the Ethics Committee of Peking University Third Hospital (License No. IRB00006761-M2016170).

### Single-cell preparation

The dissected tumor sample was digested into single-cell suspension via 1.5 mg/mL collagenase type II (Gibco, 17101015) and 1.5 mg/mL collagenase type IV (Gibco, 17104019) treatment for 30 min at 37°C. APC anti-human CD326 (EpCAM) Antibody (BioLegend, 369810) and PE anti-human CD235a (Glycophorin A) Antibody (BioLegend, 349106) and 7-AAD viability staining solution (BioLegend, 420404) were used in FACS, and CD235a-CD326+ cells were collected. For this sample, single cells were picked into PCR tubes using mouth pipette.

### Validation of structure variations in CRC cells

Insertions and deletion in CRC called from SMOOTH-seq were selected for PCR validation. PCR primers were designed such that the amplicons span the breakpoints and produce PCR products. Then, the normal tissue gDNA and GM12878 cells gDNA and PBMC gDNA from another patient were amplified with the same primers were used as control. Amplification was done by Tks Gflex DNA Polymerase. All PCR amplicons were run on 1% agarose gels.

### CCS reads generation and mapping.

We used ccs v4.0.0 (https://github.com/PacificBiosciences/ccs) with --minPasses 1 to generate CCS reads. Then, we used lima v1.10.0 (https://github.com/PacificBiosciences/barcoding) to demultiplex and trim barcoded CCS reads for each cell. Clean CCS reads were then mapped to hg38 human reference genome with alternate haplotype sequences removed, using pbmm2 v1.1.0 (https://github.com/PacificBiosciences/pbmm2) with --preset CCS. As for bulk NGS reads, we used fastp [37] for quality control and BWA-MEM [38] for mapping. Since K562 and CRC cells are female cells, reads aligned to chromosome Y were filtered.

### CNV analysis

To profile the CNVs of bulk NGS data with 1 Mb windows, we used Control-FREEC [39] v11.5 with the following parameters: ploidy=3, breakPointThreshold=0.8, and window=1,000,000. For SMOOTH-seq data, we employed the 1 Mb windows generated by Control-FREEC and calculated the ratio of reads detected in each window. Then, the mean values of each window from every K562 cell were normalized to 3 (the ploidy of K562, CRC cells were normalized to 2) by multiplying a constant. We calculated CV for each cell to evaluate the deviation of SMOOTH-seq method on profiling the CNVs, where the baseline of copy number used is the mean value of K562 single cells. More rigorously, we also calculated CV for each cell using bulk K562 copy number as the baseline. To segment DNA copy number, we used DNAcopy (Seshan VE, Olshen A. DNAcopy: DNA copy number data analysis. R package version 1.60.0.), which is based on circular binary segmentation (CBS) algorithm [40]. To quantitatively measure the performance of SMOOTH-seq CNV analysis, we then computed Pearson correlations of segmented copy numbers among the single cells and the bulk K562 samples, where bins within chromosome gaps were removed.

### SNV detection and benchmarking

To call SNVs in SMOOTH-seq data, we ran GATK [33] HaplotypeCaller v4.1.4.0 with the following parameters: --pcr-indel-model AGGRESSIVE, --minimum-mapping-quality 60, --read-filter MappingQualityReadFilter, --read-filter NotSecondaryAlignmentReadFilter, and --ploidy 1 (the coverage depth of CCS reads is less than 1×). We then filtered SNVs using GATK VariantFiltration with --filter-expression QD < 2.0.

To call SNVs in bulk NGS data, we followed the GATK best practice for hard filtering. We employed GATK BaseRecalibrator to recalibrate base quality score. Then, GATK HaplotypeCaller was run to call SNVs with a few modifications: --minimum-mapping-quality 20, --ploidy 2, and --pcr-indel-model NONE. In addition, GATK VariantRecalibrator was used to score variant quality for filtration. Subsequently, we filtered SNV calls using GATK VariantFiltration with --filter-expression QD < 2.0 || FS > 200.0 || SOR > 10.0 || MQRankSum < -12.5 || ReadPosRankSum < -8.0. To reduce false positive SNVs, we required all SNV calls to be supported by more than ten reads and the ratio of SNV call-supported reads at each location is larger than 40%.

To evaluate the SNV detection efficiency and accuracy of SMOOTH-seq, we used vcfeval [41] (https://github.com/RealTimeGenomics/rtg-tools) to benchmark multicell-supported SNVs (from one-cell-supported SNVs to ten-cell-supported SNVs) against the standard reference of NGS bulk. SNV calls from SMOOTH-seq and bulk data were considered consistent and determined as true positive calls, if they have the same alternative allele. To calculate the false positive rate of SNV calling (2.0 × 10^-5^ in the maintext) for each single cell, we followed the previous published workflow [7] by dividing the number of the false positive SNVs by the total covered bases in a single cell.

### SV detection and benchmarking

Since K562 standard structural variant set is not available, we used bulk PacBio continuous long reads (bulk TGS reads), which have shown high performance in SV detection [19], to call SVs as the benchmark. Bulk TGS reads were also mapped to hg38 with pbmm2.

To call SVs in our SMOOTH-seq and bulk TGS data, we used pbsv v2.2.2 (https://github.com/PacificBiosciences/pbsv), where the pbsv discover stage was run with GRCh38 tandem repeat annotations (https://github.com/PacificBiosciences/pbsv/blob/master/annotations/human_GRCh38_no_alt_analysis_set.trf.bed) passed with --tandem-repeats, and the pbsv call stage was run with --ccs.

To reduce false positive SV calls from cells, we used the following filtration steps. First, SV calls flagged with IMPRECISE (imprecise structural variation) or SHADOWED (CNV overlaps with or is encapsulated by deletion) were filtered, and only PASS calls were considered. Second, we required all SV calls to be supported by at least two reads. Third, we integrated SV calls from all the 91 K562 cells using SURVIVOR [42] and required an SV call supported by at least two cells. As for bulk TGS, we required all SV calls to be supported by at least four reads and the ratio of SV call-supported reads at each location is larger than 15%. Besides, we merged SV calls from both bulk TGS clones as another SV benchmark. SVs were merged if they were of the same type and the breakpoints were within 1kb. To evaluate the SV (except translocation) detection performance of SMOOTH-seq, we used Truvari (https://github.com/spiralgenetics/truvari) with -r 1000 --multimatch. For translocations, we used SURVIVOR to merge and count the matched ones, requiring the paired breakpoints were within ± 50kb of each other.

To quantify the performance of SV detection, we used precision (Precision=TP/(TP+FP)) to measure the ratio of true positives in all detected positives, as the genome coverage of a single cell is relatively low and we focus on the ratio of true positive SV detected. In addition, to further illustrate the performance of SV detection, we employed other metrics, including false positive rate (FPR), false negative rate (FNR), harmonic mean (HM), recall, and F1-score (F1), and they are defined as follows: FPR=FP/(TP+FP), FNR=FN/(TP+FN), HM=2×FPR×FNR/(FPR+FNR), Recall=TP/(TP+FN), and F1=2×Pre×Recall/(Pre+Recall), where TP, FP, and FN represent true positive, false positive, and false negative, respectively. Herein, FPR measures the ratio of false positives in all detected positives, FNR measures the ratio of actual positives which are undetected, HM is an aggregated performance score of FPR and FNR, recall measures the ratio of actual positives which are correctly detected, and F1-score is an aggregated performance score of precision and recall. For FPR, FNR, and HM, the lower, the better. For precision, recall, and F1-score, the higher, the better.

To compare the SVs identified using SMOOTH-seq and bulk TGS to previous NGS-based results [29], we used different criteria for different SV types. For deletions, we required the SVs from two methods had a reciprocal overlap larger than 10%. For insertions, the breakpoints need to be within ±1kb of each other.

To call SVs for CRC cells, we followed the methods described above. To reduce false positive SVs raised by reference genome, we filtered the overlapped SVs between CRC cells and K562 bulk TGS. After filtration, we used AnnotSV [43] for sv annotation.

### ecDNA detection

To detect ecDNA from CCS reads, we developed a custom pipeline with the following procedures. First, as ecDNA tends to show discordant alignments as illustrated by Fig. 3a, we analyzed all the aligned CCS reads and picked the CCS reads aligned to the reference genome with only two segments in a chiastic order. Second, we filtered these CCS reads by intersecting with the tandem repeats (length > 1kb) of human reference genome (http://hgdownload.cse.ucsc.edu/goldenPath/hg38/bigZips/hg38.trf.bed.gz). Third, duplications called in our previous step were utilized. Briefly, we filtered CCS reads overlapped with those four-cell supported duplications, requiring the coordinates were within ±100 bp of each other. In the end, after obtaining the ecDNAs in each single cell, we integrated these candidate ecDNAs from all the K562 cells and kept those supported by at least two cells and these ecDNAs were merged if their coordinates were within ± 50bp of each other.

## Supplementary Information

**Additional file 1: Supplementary figures (S1-S12).** Describe the CNVs, SVs, ecDNAs and SNVs detected by SMOOTH-seq.

**Additional file 2: Table S1.** Describes data information.

**Additional file 3: Table S2.** Describes ecDNAs in K562 cells.

**Additional file 4: Table S3.** Describes PCR validation.

**Additional file 5: Table S4.** Describes SVs in CRC.

**Additional file 6.** Review history.

## Data Availability

The analysis code is deposited in github (https://github.com/cyang235/Smooth-seq) [44] and in Zenodo with DOI: 10.5281/zenodo.4963186 [45]. The K562 Data have been deposited in the Sequence Read Archive (SRA) under BioSample accession: SAMN14945175 [46], and the CRC data is available from Genome Sequence Archive (GSA) for human under accession number HRA000568 [47].
