# Supplementary material for: SMOOTH-seq: single-cell genome sequencing of human cells on a third-generation sequencing platform
Source: Genome Biol. 2021 Jun 30;22:195. doi: 10.1186/s13059-021-02406-y (PMC8247186; doi:10.1186/s13059-021-02406-y)
Supplement: Supplementary file 1 — Additional file 1: Supplementary figures (S1-S12). Describe the CNVs, SVs, ecDNAs and SNVs detected by SMOOTH-seq. [file 13059_2021_2406_MOESM1_ESM.docx]

# Supplementary information

**Additional file 1:**

**Fig S1.** **Process testing of SMOOTH-seq**

**Fig S2.** **Generation of the two K562 clones.**

**Fig S3. Workflow of variations.**

**Fig S4. The heatmap of the deduced copy numbers in K562 bulk and single cell samples.**

**Fig S5. The heatmap and distribution of the Pearson correlation coefficients of CNV calling.**

**Fig S6. SVs in K562 cells.**

**Fig S7.** **Analysis of the four highest covered cells.**

**Fig S8. SVs detected by SMOOTH-seq in K562 cells.**

**Fig S9. ecDNAs in K562 cells.**

**Fig S10. ecDNA validation by Sanger sequencing.**

**Fig S11. SNV analysis.**

**Fig S12.** **Distribution of CNVs and duplications in CRC cells and the differences of PCR products between gDNA samples and reference genome.**


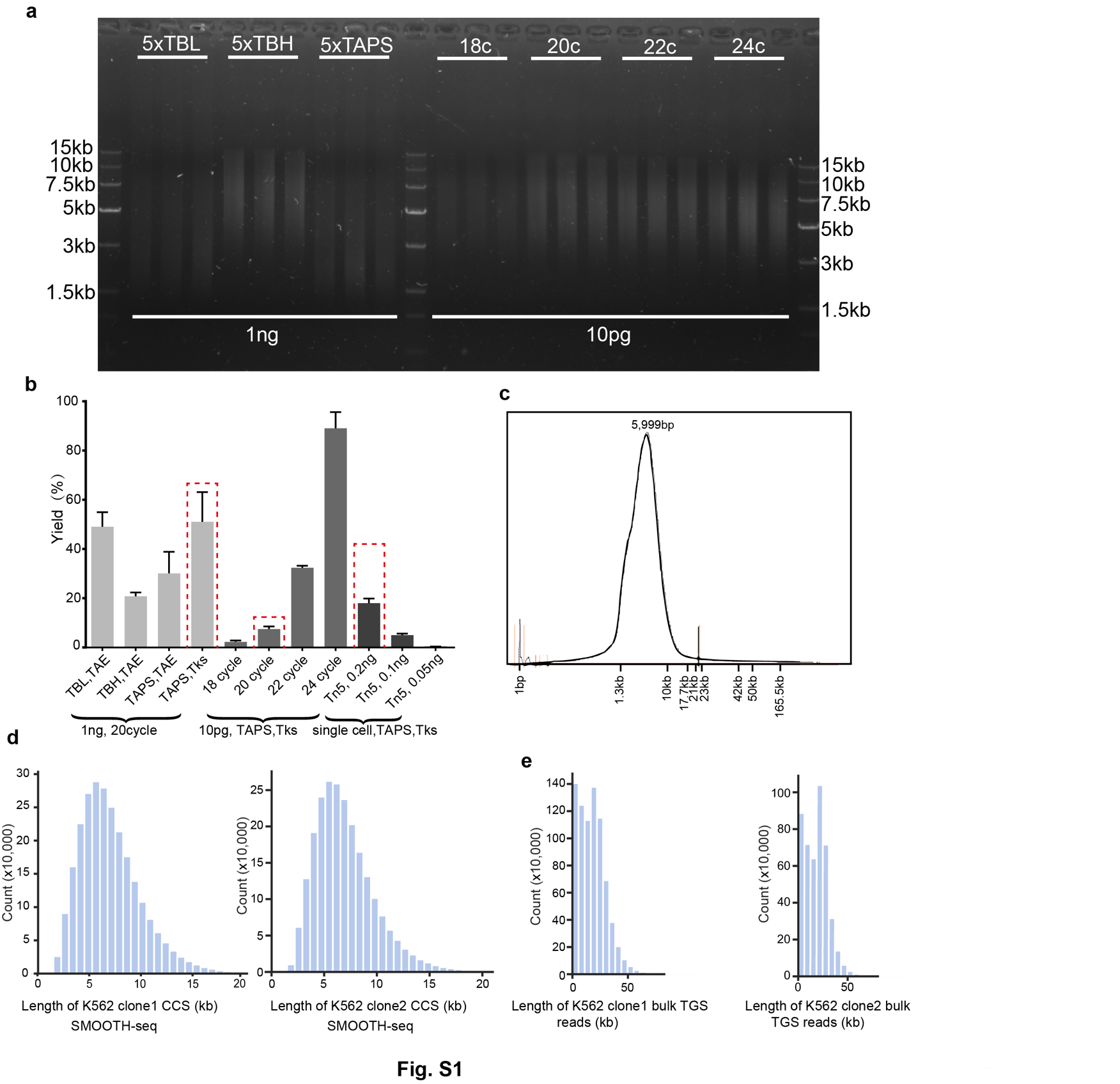


**Fig S1. Process testing of SMOOTH-seq****. a** The agarose gel showing the length distribution of PCR products of SMOOTH-seq. 5×TBL and 5×TBH are buffered supplied in the commercialized Tn5 transposase, 5×TAPS buffer was self-made as described in the methods part. The left shows the products amplified from 1ng K562 gDNA using TAE polymerase for 20 cycles. The right shows the lengths of amplicons from 10pg K562 gDNA using TAE polymerase. Running 22 or more PCR cycles shows an amplification bias for shorter fragments. **b** Relative yields of amplifying K562 gDNA and single cells by different transposon reaction buffer (TBH, TBL and TAPS), amplification polymerases (TAE and Tks), different equivalent of Tn5 transposase (0.05ng, 0.1ng and 0.2ng) and different PCR cycles from 18 to 24. In single cells the yields of amplicons by Tks are higher with 0.2ng Tn5 when amplified for 20 cycles. **c** The length distribution of amplicons using 0.2ng Tn5 transposes for fragmentation and Tks for amplification with 20 cycles from a single cell. The products enriched at around 6kb. **d** The length distributions of SMOOTH-seq CCS reads in sequencing clone 1 or clone 2 K562 cells. **e** The length distributions of bulk TGS reads in sequencing clone 1 or clone 2 K562 cells.


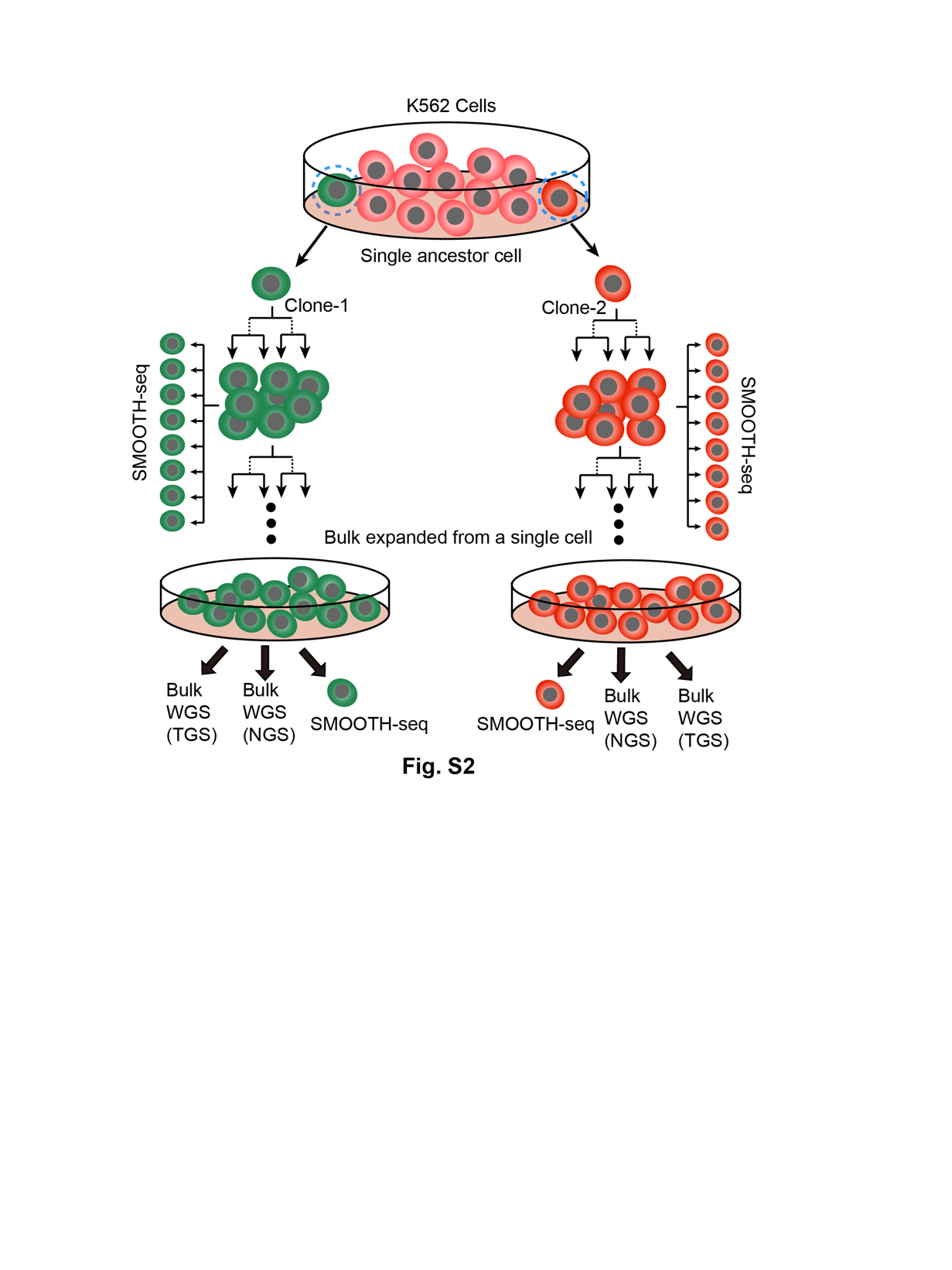


**Fig S2. Generation of the two K562 clones.** The experimental scheme showing the generation of two independent K562 clones. The daughter cells during early passages and late passages of these two clones were analyzed using SMOOTH-seq and the bulk WGS was performed using the about one million cells to extract the gDNA.


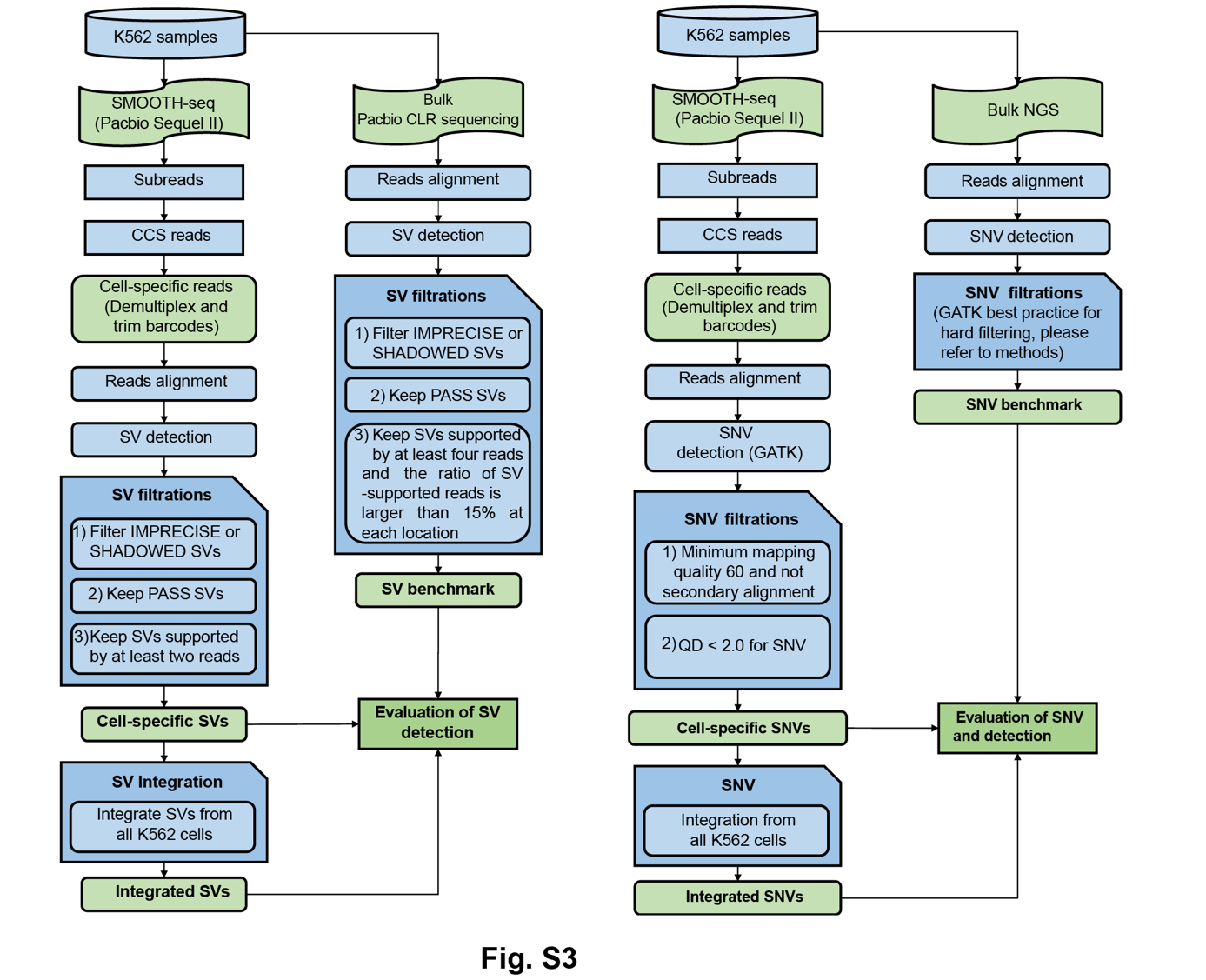


**Fig S3. Workflow of variations.** The analysis workflow of variations (including SVs and SNVs) in K562 samples.


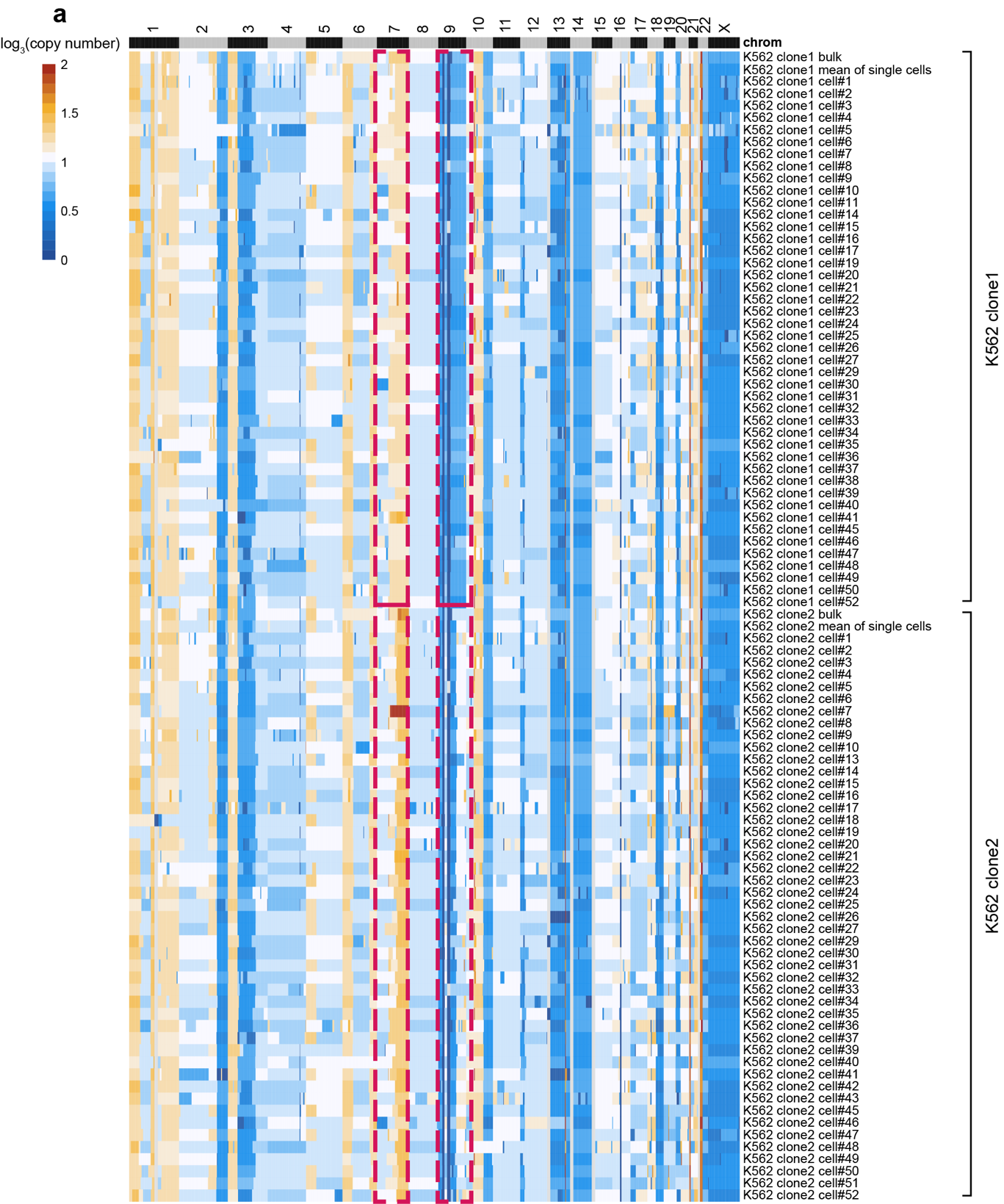


**Fig S4. The heatmap of the deduced copy numbers in K562 bulk and single cell samples.** The Red dotted frame highlights the differences of CNVs on the long arm of chromosomes 7 and 9 between these two clones.


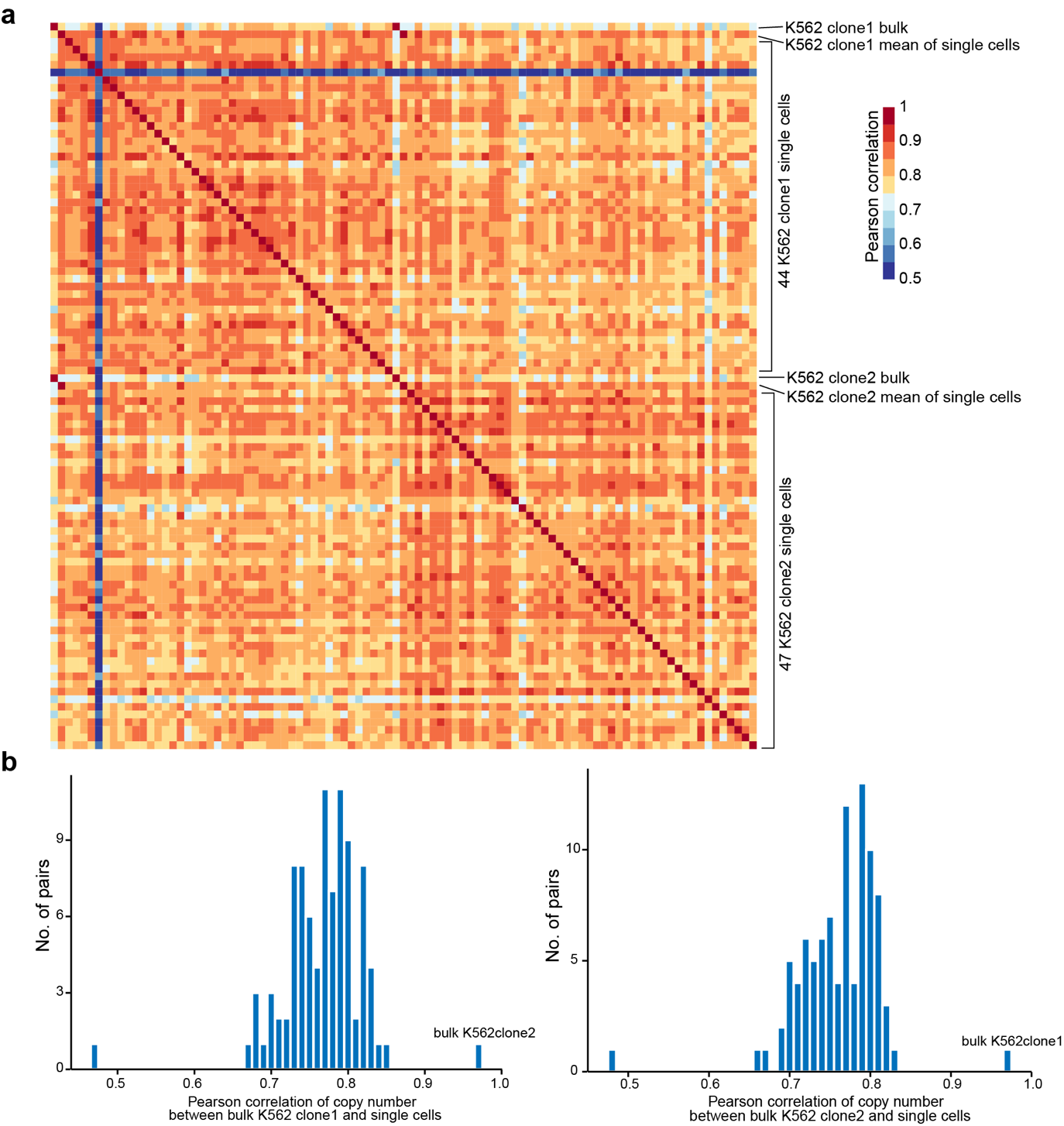


**Fig S5. The heatmap and distribution of the Pearson correlation coefficients of CNV calling.**

**a** The heatmap of the correlation coefficients of CNV calling among the single cells and the bulk K562 samples. **b** Pearson correlations of segmented copy numbers among the single cells and the bulk K562 samples (*P*-values < 1×10^-5^).


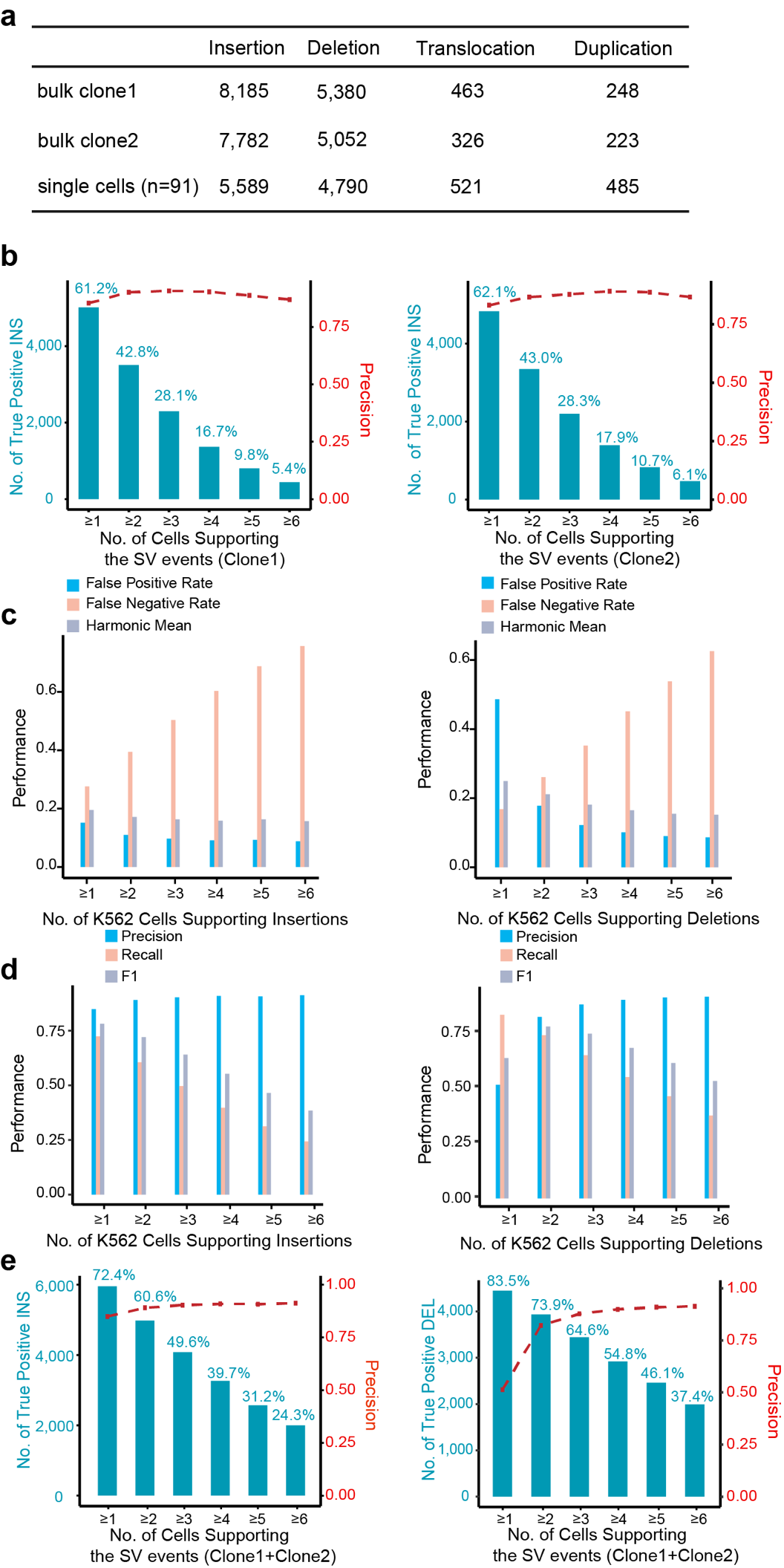


**Fig S6. SVs in K562 cells. a** An overview of SVs detected by bulk TGS in clone 1, clone 2 K562 cells and by SMOOTH-seq in K562 cells. **b** Precision of SMOOTH-seq detecting insertions and the percentage of true positive insertions with different numbers of supporting clone 1 or clone 2 K562 cells. **c** The false positive rate (FPR) and false negative rate (FNR) of multicell-supported insertions and deletions. FPR=FP/(TP+FP), FNR=FN/(TP+FN), Harmonic Mean is a composite measure used as an aggregated performance score of FPR and FNR. **d** The Precision, Recall and F1-score of multicell-supported insertions and deletions. Precision=TP/(TP+FP), Recall=TP/(TP+FN), F1=2×Pre×Recall/(Pre+Recall). **e** Precision of SMOOTH-seq detecting deletions and insertions the percentage of true positive deletions and insertions with different numbers of supporting K562 cells from merged clone 1 and clone 2.


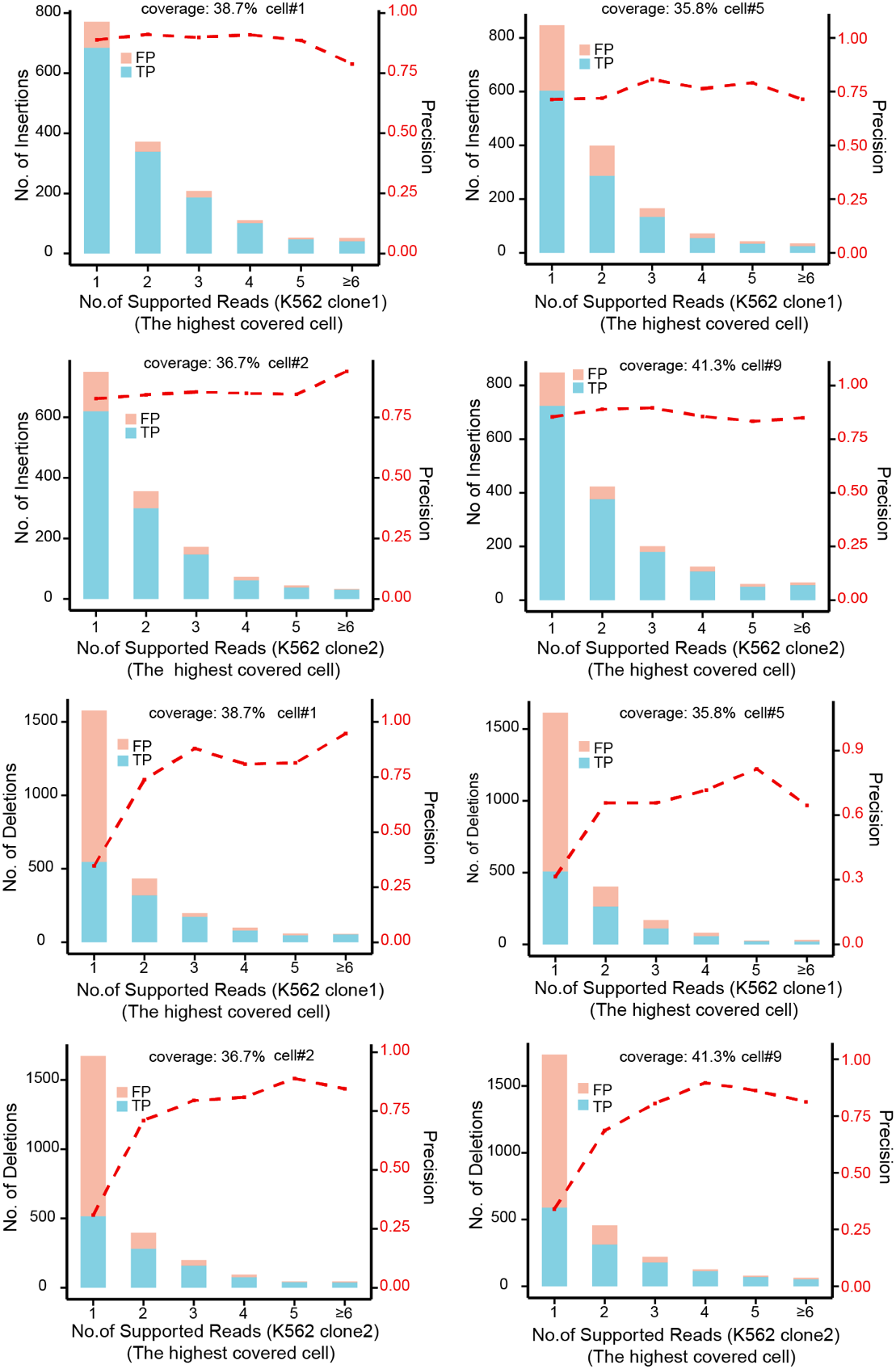


**Fig S7. Analysis of the four highest covered cells.** The precision and true positive numbers of detected insertion and deletion events under different supporting reads in four single K562 cells with highest coverage.


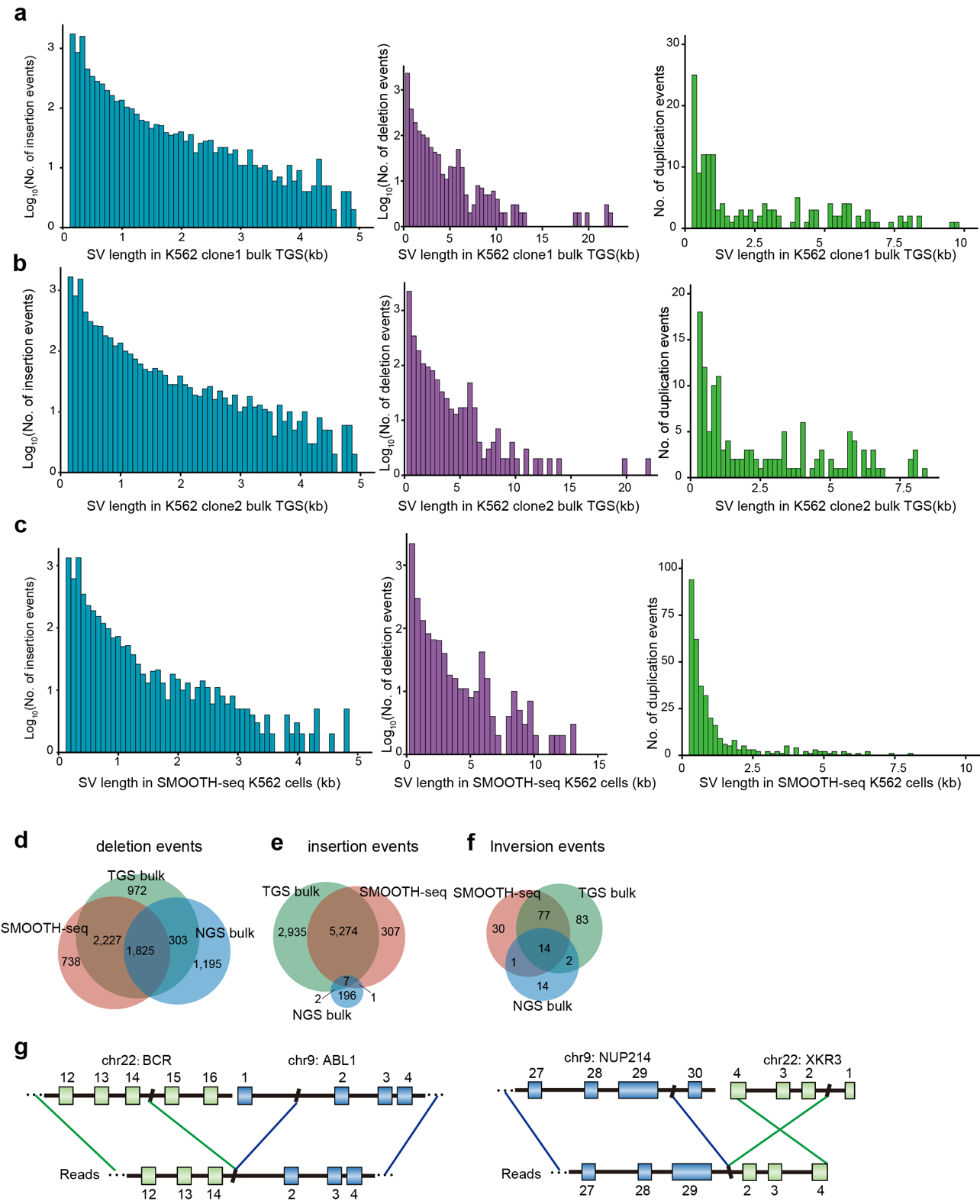


**Fig S8. SVs detected by SMOOTH-seq in K562 cells. a,b** Histograms of length distributions for deletion, insertion and duplication events detected by bulk TGS in clone 1 and clone 2 K562 cells. **c** Histograms of length distributions for deletion, insertion and duplication events detected by SMOOTH-seq in K562 cells. **d** Overlapping between deletion events detected by SMOOTH-seq, bulk TGS and reference bulk NGS. **e** Overlapping between insertion events detected by SMOOTH -seq, bulk TGS and reference bulk NGS. **f** Overlapping between inversion events detected by SMOOTH-seq, bulk TGS and reference bulk NGS. **g** Detailed translocation events to cause classical fusion genes: the 14^th^ intron of BCR gene on chromosome 22 linked with the 1^st^ intron of ABL1 gene on chromosome 9 results in a fusion transcript; translocation of the 29^th^ intron of NUP214 gene on chromosome 9 to the 1^st^ intron of XKR3 gene on chromosome 22 results in a fusion transcript.


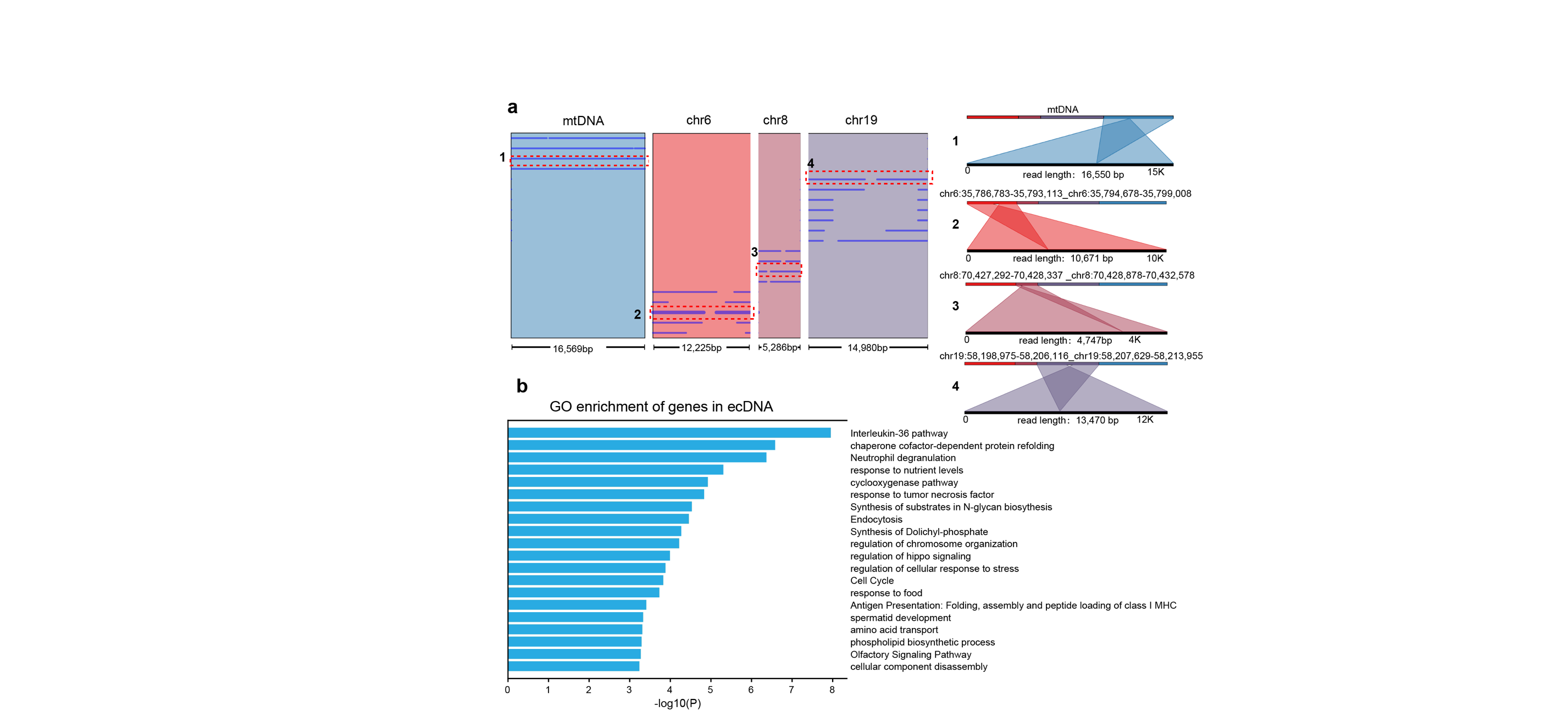


**Fig S9. ecDNAs in K562 cells. a** Ribbon (https://github.com/MariaNattestad/ribbon) showing examples of detected circular DNAs in K562 cells. Mitochondrial DNA was shown as positive control and nearly full length of ecDNA was captured. ecDNAs with different CCS reads spanning the circulation sites are shown. 1-4 show the specific CCS reads and their mapping to the genome region. **b** The top GO terms for genes inside ecDNA regions.


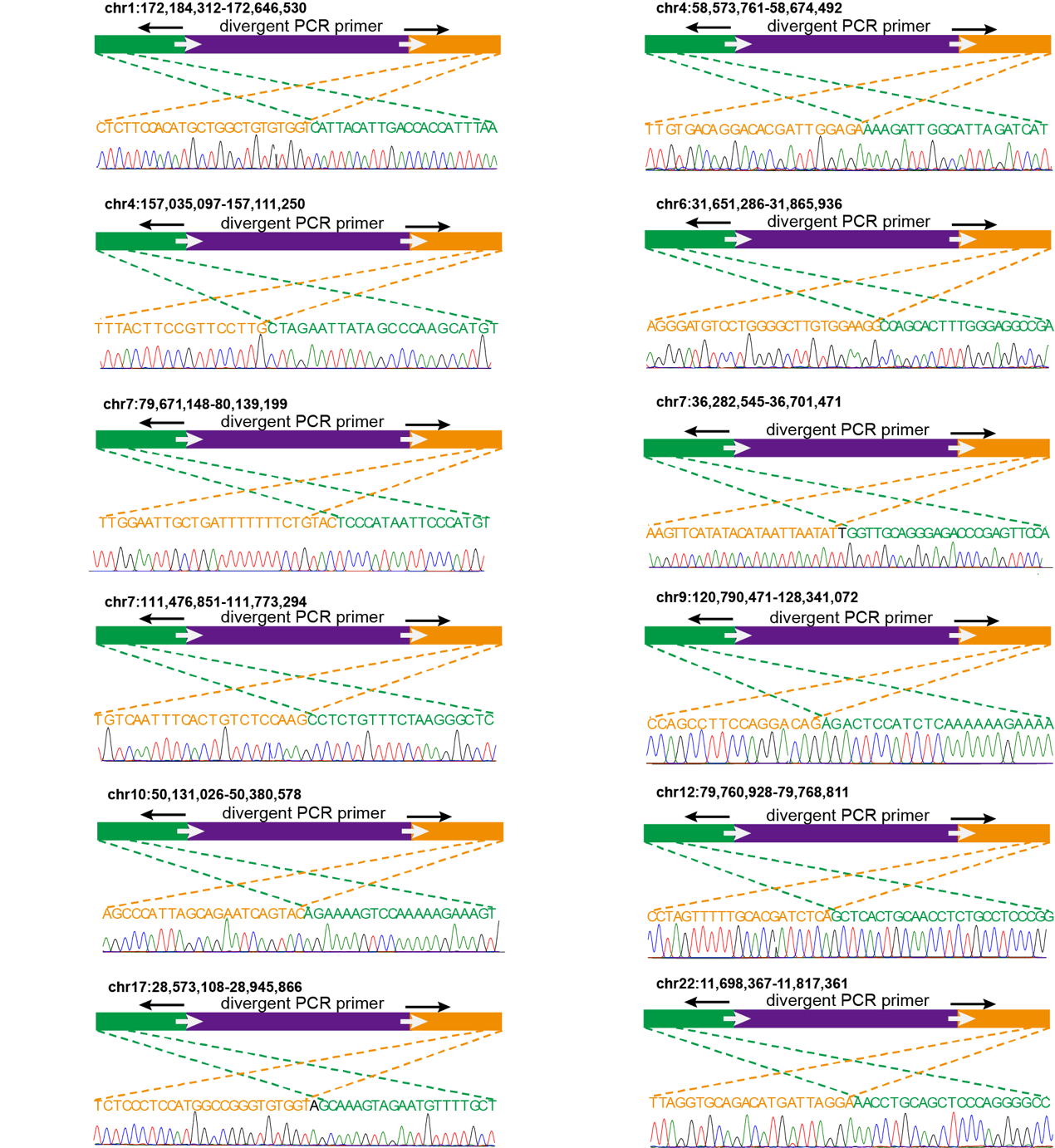


**Fig S10. ecDNA validation by Sanger sequencing.** Sanger sequencing validation of circulization sites of 12 candidate ecDNA in K562 cells identified using SMOOTH-seq. Sequencing results show a reversed order in genome as expected.


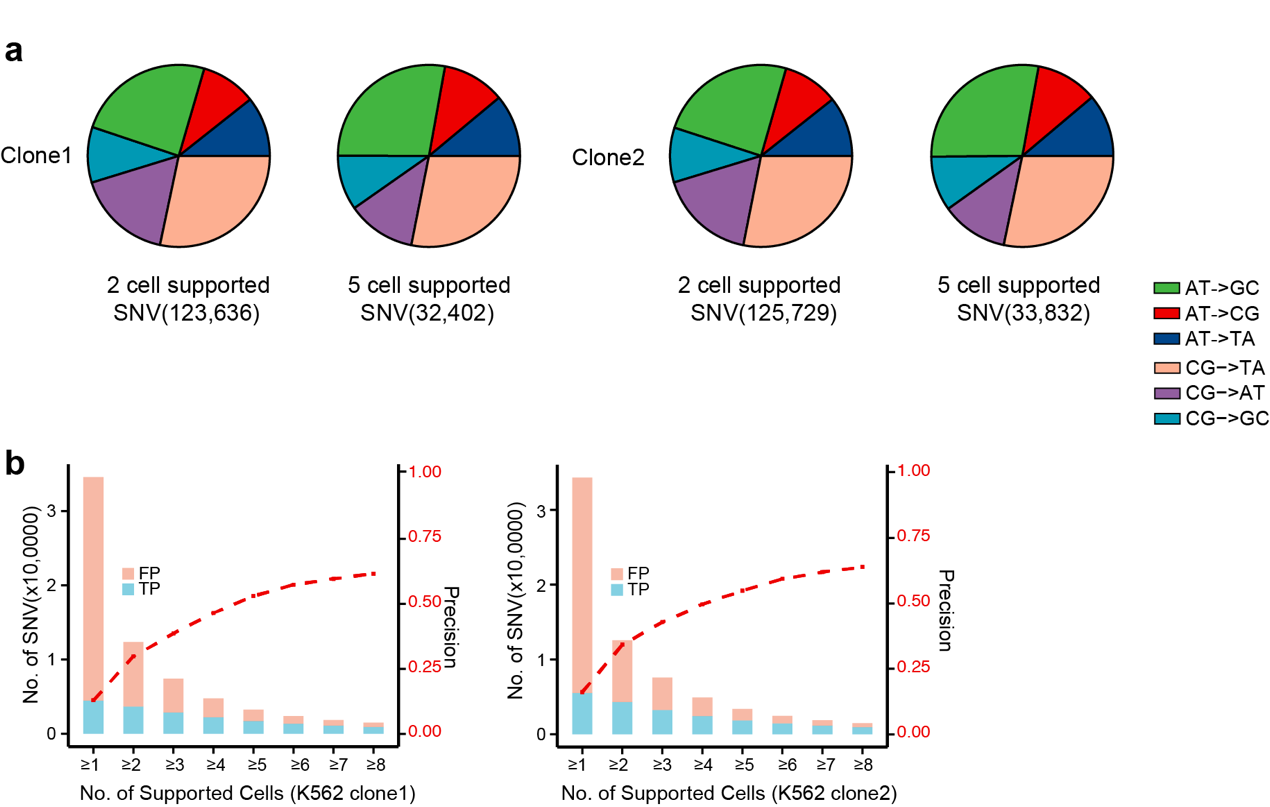


**Fig S11. SNV analysis.** **a** Spectra of SNVs supported by at least 2 and at least 5 K562 cells in clone 1 and clone 2. **b** The precision and numbers of true positive and false positive events for detected SNV under different number of supporting cells for single K562 cells.


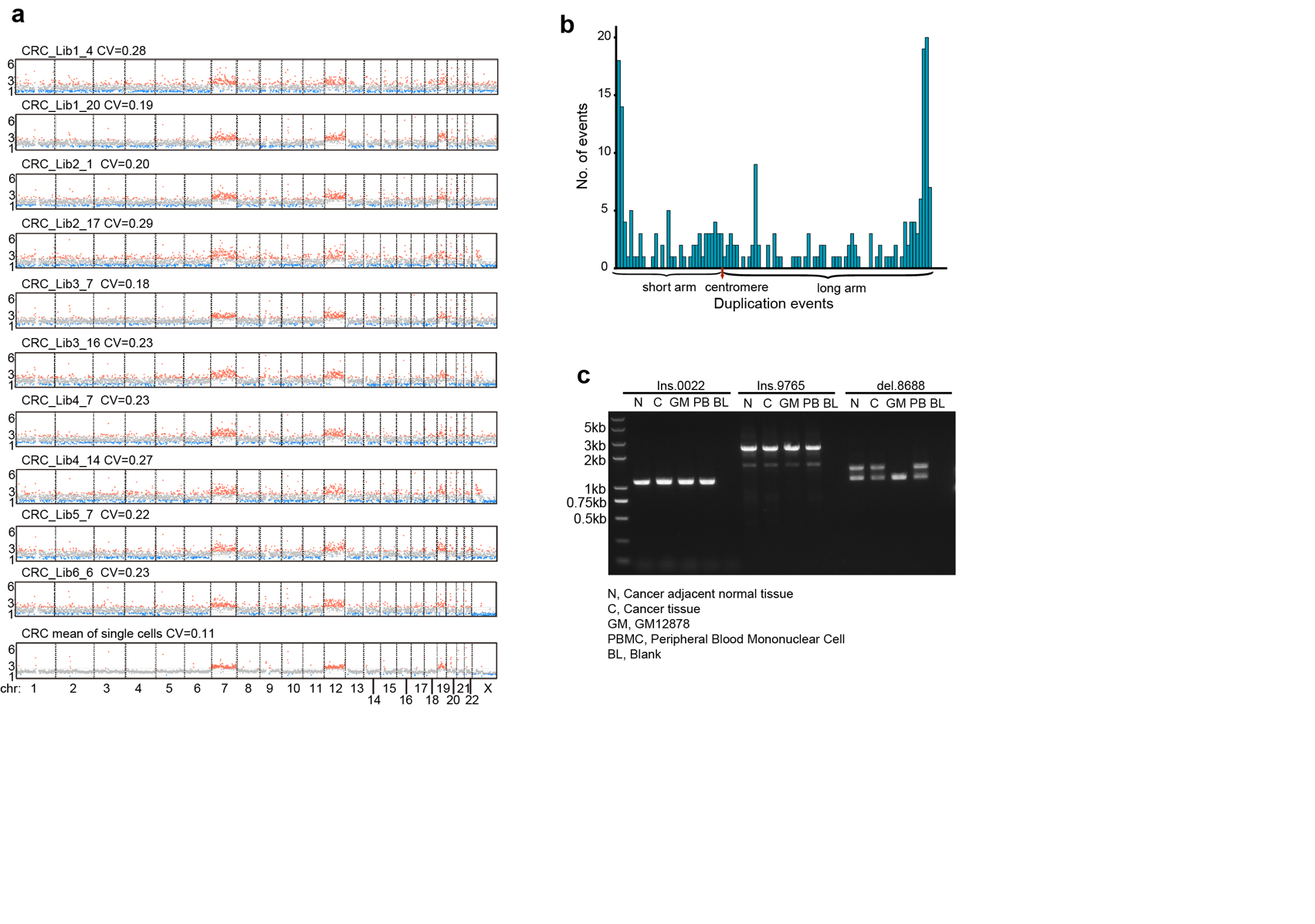


**Fig S12.** **Distribution of CNVs and duplications in CRC cells and the differences of PCR products between gDNA samples and reference genome.** **a** CNVs of single CRC cells showing in 1Mb windows. Digitized copy numbers across the genome are plotted in representative single CRC cells. The mean copy numbers are the averages of 24 cells from CRC cells. **b** Distribution of duplication events on the chromosomes. The chromosomes are separated into 100 windows from the centromere to the telomere. The number of duplication events are calculated in each window. The uniform distribution test was conducted and the *P*-value was less than 1×10^-3^. **c** PCR validation of insertions and deletions events existing in cancer tissue, cancer adjacent normal tissue, GM12878 and Peripheral Blood Mononuclear Cell. BL, Blank.
