# Supplementary material for: SMOOTH-seq: single-cell genome sequencing of human cells on a third-generation sequencing platform
Source: Genome Biol. 2021 Jun 30;22:195. doi: 10.1186/s13059-021-02406-y (PMC8247186; doi:10.1186/s13059-021-02406-y)
Supplement: Supplementary file 6 — Additional file 6. Review history. [file 13059_2021_2406_MOESM6_ESM.docx]

Review History

**First round of review**

**Reviewer 1**

**Were you able to assess all statistics in the manuscript, including the appropriateness of statistical tests used?**

Yes: statistics looks fine.

**Were you able to directly test the methods?**

No

**Comments to author:**

The manuscript by Fan et al demonstrated single cell whole genome long read sequencing. As emphasized by the authors, long reads are more powerful in revealing structural variations. The manuscript serves as a good example of technical demonstration and a new type of resource. I only have some minor concerns regarding potential application of the strategy: since the whole genome sequencing was performed with a low depth (0.4x) and low genome coverage (19%), how can we compare the variations among single cells? How would the requirement of multiple cells with the same variation affect false positive and false negative rates? I think the technical demonstration part is clear but a more thorough discussion is important.

**Reviewer 2**

**Were you able to assess all statistics in the manuscript, including the appropriateness of statistical tests used?**

Yes: I reviewed the statistical analysis results.

**Were you able to directly test the methods?**

No

**Comments to author:**

Fan et al introduce Smile-seq, a single-cell DNA sequencing method that uses low concentration of Tn5 loaded with one type of adapter sequence to isolate kilobase-scale fragments from single-cell genomes suitable for PacBio HiFi sequencing. Achieving long-read sequencing in single cells represents an important technical goal, so the article's topic is interesting and relevant. Use of Tn5 is common, but using lower concentration to generate longer fragments for PacBio sequencing is new.

Three datasets are examined: analysis of clonal and subclonal mutations in K562, identification of ecDNA, and analysis of a patient cancer sample. First, the authors show that per-clone precision of most major classes of SVs is between 25% (for duplications and translocations) and ~80% (for indels), with 60-70% precision for SNVs. Experimental follow-up of 8 insertions and recapitulation of three K562 translocations suggests an overall ~50% PCR validation rate for SVs. Second, the authors show that Smile-seq can identify a wide range of ecDNAs from K562, particularly by reading through the ecDNA junction, with a 90% PCR validation rate. Finally, the authors analyze a patient colorectal cancer sample for SVs, with 5 of 8 candidate patient-specific SVs successfully validated.

Unfortunately, the analysis for benchmarking the performance of each variant type remains insufficient, leaving many questions about how many of the variants in a given single cell are artifactual or biologically real. A much more rigorous and thorough analysis is needed.

Major points

1. The current "benchmarking" gives various statistics, e.g., the size of variants, how many are found with support from how many cells. Then a small number of calls are validated, with mixed success rates, from PCR. But the level of analysis falls far below the standard for a variant calling methods paper. For example, even though one's interest is in somatic variants, germline variants should be studied in great detail first, so that you can take advantage of a gold standard set of variants. This seems like a basic task, but the authors only consider somatic variants.

2. Copy number analysis consists of a series of accuracy estimates without even describing how accuracy was measured. From Fig 1b, it seems that the profile has a substantial level of noise, making CNV analysis difficult. Some basic QC analysis and a more careful CNV analysis is needed.

3. Because the cells were sequenced at such a low coverage, it is hard to interpret the accuracy estimates. For the purpose of benchmarking, higher coverage data would have been helpful to separate the impact of sequencing depth.

4. As an example, I will describe some of the issues with SNV/indel analysis. Although current estimates of PacBio CCS put the error rate at around 0.5% or less with proper error correction and consensus calling from multi-pass CCS data, the rate of erroneous indels and SNVs remains around 10-15% in raw data (Wenger et al 2019). Additionally, the length distribution of deletions (mostly <1-kb long) raises the concern that these deletions might represent sequencing errors. The authors do not present a clear analysis of how they reduce erroneous SNV and indel calls from long-read data. Specifically, the authors need to demonstrate that per single cell, they can identify and rule out SNV and <1kb-long indels that might originate from errors during sequencing. Four pieces of data from the authors suggest that many SNV, indel, and SV calls per single cell from their analysis might be errors.

a. First, in Figure 2c, the authors show how the percent of true-positive deletions drops off as more cells are required to share them. The near-linear decay in the percentages suggest that the number of deletions shared amongst a group of cells arises from random sampling (for example, a single cell has 71% of its deletions as true-positives, but the percent of deletions shared in two cells being positive suggests a product between the percentages of two independent cells, 0.716 * 0.716 = 0.516, which is close to the reported 55.2%). If the authors were capturing true clonal structure, then the decay in the percentage of shared deletions would deviate from the linear trend. The percentages do deviate from products of independent probabilities as more cells are required to support an event, but the trends for smaller groups of single cells suggest that many individual deletion calls per single cell are erroneous that require consensus calling to filter.

b. Second, in the patient CRC sample, the authors find that 60% of SVs called from the CRC sample were shared in K562, and although the authors do focus on CRC-specific SVs, the high percentage suggests that the analysis of Smile-seq does not sufficiently filter out false-positive SV calls. The description of the PCR results in gDNA suggests that several SVs were found in all gDNA samples albeit at different sizes, suggesting false-positive mutation calls.

c. Third, in the SNV analysis, it is not clear how the authors derived a false-positive rate of 2e-5, so clarification of how the authors estimated the false-positive rate is necessary. On the other hand, a back-of-the-envelope calculation based on the data suggests that the false discovery rate for SNVs per single cell might be higher than the authors estimate. Less than 20% of bulk-defined SNVs were captured in individual cells, suggesting that approximately 3,000 SNVs called per cell are either cell-specific or erroneous. This would roughly translate to about 17,000 total non-bulk SNVs per single-cell (if we assume that at most 17% of the cell's true SNVs are captured). These numbers would place the overall single-cell mutation frequency at approximately 35 mutations/Mb, which is seemingly high for K562 (and, based on MutSigCV analysis presented in Lawrence et al Nature 2013, this estimated rate would lie in the far-upper-tail of mutation rates for leukemias). Additionally, if the estimated precision per single cell for SNVs is approximately 10-20% (based on their cell-sharing analysis), then 80-90% of the mutation calls are likely to be false discoveries, which would suggest that most of the excess, non-bulk SNVs identified per single cell are likely false positives.

d. Fourth, the authors report a consistent validation rate for insertions and SVs of ~50% (4 of 8 for K562 insertions, 1 translocation reported, and 5 of 8 insertions in CRC). Many of the events selected for experimental follow-up may not be prevalent enough across the clones to be well-validated, but the rate suggests that the authors' callset may contain many false or erroneous events.

5. The authors should describe their quality control strategies in more detail or implement a workflow for distinguishing false-positive SNVs and indels per cell. The analysis of cell sharing is a good starting point, but the currently presented analysis does not sufficiently address how SNVs called from long reads even before cell-sharing analysis and at the step of read handling. The authors have not shown how these candidate mutation calls are separated into likely-false and likely-true calls, how the multiple passes or overlapping molecules are used to remove false SVs, and how <1kb-long deletions are sifted. The <1kb-long deletions are concerning because this represents a known error mode for long-read sequencing.

6. The authors do not establish how true SVs can be distinguished from false-positive SVs from low-coverage (average of 0.4x) single cell data. This problem is distinct from whether single cells can re-identify SVs from a bulk WGS sample of an ancestral or sibling clone. The latter problem is ultimately a measure of sensitivity, while the former problem is one of precision. The cell-sharing analysis provides an indirect sense of how many deletions might be false, but it is not clear how the authors filter out poor SVs from their initial call set before conducting cell-sharing analysis. Although the authors do use 10-pass CCS which should theoretically bring down the false-positive rate, the authors have not established whether filtering out erroneous bases is possible with sparse coverage. Furthermore, the lower coverage suggests that many events captured in one cell cannot be corroborated in another cell, since it is likely that not all loci will have at least one fragment for all cells. Ideally, the authors could sequence a portion of their cells at much higher overall coverage to show how many SVs are supported by separate long read fragments (and not just from multiple passes of the same CCS molecule).

7. No discussions of inversions are provided. Past phased whole-genome analysis of K562 (Zhou et al Genome Biology 2019; Dixon et al Nature Genetics 2018) show the presence of many large inversions (63kb and 125kb inversions, for instance). Given that long reads have distinct advantages over short-read sequencing in resolving single or consecutive inversion events, we highly recommend an analysis of inversions from the K562 clones.

8. The draft contains grammar and spelling errors that require careful proofreading.

**Authors Response**

**Point-by-point responses to the reviewers’ comments:**

*Thank the reviewers for appreciating the significance of our work! We have considered all of the reviewers’ comments carefully and have made corresponding revisions which were marked in yellow in the revised version of our manuscript*

*The third-generation sequencing (TGS) based genome sequencing method using bulk samples is now well-established technology [1, 2]. It has been systematically evaluated and is approved to be able to accurately detect genomic structural variations (>50bp) owing to the long read length, especially for Pacbio platform [3]. So we used genome sequencing of bulk samples on the Pacbio platform as gold standard to evaluate our smile-seq method for SV detection. We aligned our TGS-based single-cell and bulk sequencing reads directly to the reference genome (hg38), and compared the variants called in the Smile-seq dataset to those called in TGS bulk samples to evaluate the efficiency and accuracy of Smile-seq. According to that, our method was shown to having high efficiency and accuracy in detection of deletion and insertion events when requiring at least two single cells supporting the SV events.*

*As a complement, we also picked 8 insertion events in CRC to perform PCR validation, and 5 of 8 insertions had validated successfully. Since only 8 out of 1,376 insertion events were chosen, the ‘validation ratio’ (5/8) cannot be used to estimate the true positive or false positive rates. We think it is reasonable to use genome sequencing of bulk samples on the Pacbio platform as gold standard to estimate the true positive and false positive rate and evaluate our smile-seq methods for SV detection.*

*It is well known that the main purpose of developing single cell sequencing technology is to increase the sensitivity of corresponding bulk samples sequencing technology. In this way, single cell sequencing technologies have two major advantages compared to corresponding bulk samples sequencing technologies:*

*(1) As rare as one or two individual cells can be analyzed now, instead of hundreds of thousands of cells are needed as starting materials;*

*(2) The heterogeneity and subpopulations within tissue or a population of cells can be revealed now, which is usually masked in bulk sample sequencing results.*

*Except these, the single cell sequencing technology cannot surpass bulk sample sequencing technology. For example, it is well known that TGS-based genome sequencing methods usually have high false positive rate for SNV and indel detection (this is partially due to the fact that some of the sequencing errors of TGS based platform are not really random [4] and cannot be simply removed by sequencing many more times). TGS-based single cell sequencing technology will not improve the accuracy of SNV and indel detection in general. So in our paper we did not try to surpass TGS-based genome sequencing for the accuracy of SNV and indel detection.*

Reviewer #1: The manuscript by Fan et al demonstrated single cell whole genome long read sequencing. As emphasized by the authors, long reads are more powerful in revealing structural variations. The manuscript serves as a good example of technical demonstration and a new type of resource. I only have some minor concerns regarding potential application of the strategy: since the whole genome sequencing was performed with a low depth (0.4x) and low genome coverage (19%), how can we compare the variations among single cells?

How would the requirement of multiple cells with the same variation affect false positive and false negative rates? I think the technical demonstration part is clear but a more thorough discussion is important.

*Response: Thank the reviewer for appreciating the importance of our study. We believe that Smile-seq would be a powerful technique for single cell genome analysis, as it generates reads of kilobase-long instead of just several hundred base pairs (around 6kb vs around 0.6kb). While on the other hand, we admit the relatively low coverage of the genome by Smile-seq because of the still very high cost of the third-generation sequencing platform, especially for the Pacbio Sequel II platform. It generated highly accurate circular continuous consensus (CCS) reads by correcting multiple passes. Meanwhile, the total CCS reads of a flowcell were only about 15G-20G, corresponding to 16 single cells in our study. In this case, each cell cost about $260 (one run cost about $4,000 for 16 single cells). Yet the high cost of TGS sequencing prevented us from sequencing for much higher depth.*

*In fact, current NGS platform-based scWGS methods (such as MDA, MALBAC, DOP-PCR) usually cover about 15%-20% of the genome with 1Gb data [5, 6], indicating that Smile-seq showed similar sensitivity as NGS platform-based scWGS methods when sequencing at the same depth for an individual cell. In most cases of single cell genome sequencing (NGS platform-based), the researchers sequenced single cells with relatively shallow coverage and called features by requiring the events from two or more individual cells (For example, call a SNV by requiring that it is detected in two or three single cells to remove false positive calls). The shared features were extracted and used to evaluate the variations among single cells. Similarly, we can compare the heterogeneity of different structure variations by calculating the ratios of supporting cells to the total number of cells covered the structure variation sites.*

*For two different clones of cancer cells in the same patient, if there is only one differential SNV mutation between them, it is very difficult for NGS-based scWGS methods to discriminate between the clones due to allele dropouts. Only when there are ten or more differential SNVs between two cancer clones, it is routine for NGS-based scWGS methods to discriminate between the clones. Similarly, if there is only one differential SV between two different clones of cancer cells, it will be very difficult for Smile-seq to discriminate between the clones. But if there are ten or more differential SVs between two cancer clones, it is relatively easy to discriminate between the clones under the current sequencing depth (20% genome coverage). Since we detect several thousand SV events in a CRC patient, it is quite possible that there will be ten or more differential SV events between two different clones of cancer cells in the same patient.*

*We understand that researchers will be interested in false positive and false negative rates (FPR and FNR) of multicell-supported SVs, therefore we have plotted Figure R1a for illustration, where FPR=FP/(TP+FP), FNR=FN/(TP+FN), and HM is the harmonic mean of FPR and FNR. Herein FPR measures the ratio of false positives in all detected positives, FNR measures the ratio of actual positives which are undetected, and HM is an aggregated performance score of FPR and FNR. For all the above metrics, the lower, the better. As displayed in Figure R1a, FPR and HM show a downtrend from at least one-cell-supported to at least multicell-supported for both deletions and insertions, while FNR uptrend. Compared to the FPR of at least one-cell-supported deletions (48.6%), the FPR of at least two-cell-supported deletions drops sharply to 17.8%, indicating the efficiency of cell-sharing strategy. Besides, as illustrated in Figure R1b, the number of SVs decreases if increasing the number of supporting cells for each SV. Actually, by requiring an SV supported by at least two cells, there will be a balance between the accuracy of SV detection and the number of SV detected. Therefore, we chose the SV events supported by at least two single cells, which is in consistent with our previous analysis. We have added the results in Fig. 2 and Fig. S4, and discussed more about how we made the criteria when doing the single cell SV analysis in the revised manuscript.*

*In the previous version of our manuscript, we used “Precision” (Pre=TP/(TP+FP), measuring the ratio of true positives in all detected positives to quantify the performance of SV detection, as the genome coverage of a single cell is relatively low and we focus on the ratio of true positive SV detected. Through integrating multiple cells, we can increase the overall genome coverage and evaluate the impact of multicell support on calling structural variations. To pair Precision, we utilize another two metrics, Recall and F1-score, and they are defined as follows: Recall=TP/(TP+FN), F1=2×Pre×Recall/(Pre+Recall), where Recall measures the ratio of actual positives which are correctly detected, and F1-score is an aggregated performance score. For Pre, Recall and F1, the higher, the better. Herein, by definition, Precision is equal to 1-FPR, and Recall 1-FNR. We have added Figure R1c as another illustration of multicell-supported SV detection performance, which is consistent with Figure R1a.*

*Figure R1: The performance of Smile-Seq detecting multicell-supported SVs. (a) The false positive rate (FPR) and false negative rate (FNR) of multicell-supported insertions and deletions. FPR=FP/(TP+FP), FNR=FN/(TP+FN), Harmonic Mean is an aggregated performance score of FPR and FNR. (b) The precision of SV detection and the number of SV detected under different numbers of supporting cells. (c) The Precision, Recall and F1-score of multicell-supported insertions and deletions. Precision=TP/(TP+FP), Recall=TP/(TP+FN), F1=2×Pre×Recall/(Pre+Recall).*

Reviewer #2: Fan et al introduce Smile-seq, a single-cell DNA sequencing method that uses low concentration of Tn5 loaded with one type of adapter sequence to isolate kilobase-scale fragments from single-cell genomes suitable for PacBio HiFi sequencing. Achieving long-read sequencing in single cells represents an important technical goal, so the article's topic is interesting and relevant. Use of Tn5 is common, but using lower concentration to generate longer fragments for PacBio sequencing is new.

Three datasets are examined: analysis of clonal and subclonal mutations in K562, identification of ecDNA, and analysis of a patient cancer sample. First, the authors show that per-clone precision of most major classes of SVs is between 25% (for duplications and translocations) and ~80% (for indels), with 60-70% precision for SNVs. Experimental follow-up of 8 insertions and recapitulation of three K562 translocations suggests an overall ~50% PCR validation rate for SVs. Second, the authors show that Smile-seq can identify a wide range of ecDNAs from K562, particularly by reading through the ecDNA junction, with a 90% PCR validation rate. Finally, the authors analyze a patient colorectal cancer sample for SVs, with 5 of 8 candidate patient-specific SVs successfully validated.

Unfortunately, the analysis for benchmarking the performance of each variant type remains insufficient, leaving many questions about how many of the variants in a given single cell are artifactual or biologically real. A much more rigorous and thorough analysis is needed.

*Response: Thank the reviewer for appreciating the significance and novelty of our study. We developed Smile-seq and validated the performance of this method using two clones of K562 cells and an in vivo human colorectal cancer sample. The third-generation sequencing (TGS) based genome sequencing method using bulk samples is now a well-established technology [1, 2]. It has been systematically evaluated and is approved to be able to accurately detect genomic structural variations (>50bp) owing to the long read length, especially for Pacbio platform [3]. So we used genome sequencing of bulk samples on the Pacbio platform as gold standard to evaluate our Smile-seq method for SV detection. To make it clear how we decide the precision of variations called in each cell/clone, we added detailed analysis workflow in the revised manuscript (also see Figure R2 in below). We added detailed discussions about how we filtered the false positive calls in a single cell as suggested by the reviewer. We believe that Smile-seq is a significant breakthrough in scWGS, which showed clear advantages in SV analysis.*

*Figure R2: The analysis workflow of genomic variations (including SVs, SNVs and Indels) in K562 samples.*

1. The current "benchmarking" gives various statistics, e.g., the size of variants, how many are found with support from how many cells. Then a small number of calls are validated, with mixed success rates, from PCR. But the level of analysis falls far below the standard for a variant calling methods paper. For example, even though one's interest is in somatic variants, germline variants should be studied in great detail first, so that you can take advantage of a gold standard set of variants. This seems like a basic task, but the authors only consider somatic variants.

*Response: Sorry that we might not make it clear that the variants we called in K562 cells, which we used to clarify our methods, were actually germline variants. They were originally somatic mutations for the patient from whom K562 cell line was derived. But after being cultured in vitro and becoming a cell line, these genomic variants behave as ‘germline’ ones for the cell line, especially when we used a single-cell derived pure cell clone (We put one individual cell in a well of 96-well plate and expanded it to millions of cells in culture). We aligned our single-cell and bulk sequencing reads directly to the reference genome (hg38), and the variants called in the third-generation sequencing (TGS) bulk samples were taken as gold standard to analyze the efficiency and accuracy of Smile-seq. The third-generation sequencing using bulk samples is widely accepted as gold standard for other analyses in the literature [1-3]. Specifically, we used “Precision” (Pre=TP/(TP+FP), measuring the ratio of true positives in all detected positives to quantify the performance of SV detection, as the genome coverage of a single cell is relatively low and we focus on the ratio of true positive SV detected.*

*For PCR validation part, we picked 8 insertion events to perform PCR validation as a complement, and 5 of 8 insertions had validated successfully. Since only 8 out of 1,376 insertion events were chosen, the ‘validation ratio’ (5/8) cannot be used to estimate the true positive or false positive rate. We think it is reasonable to use genome sequencing of bulk samples on the Pacbio platform as gold standard to estimate the true positive and false positive rate and evaluate our smile-seq methods for SV detection.*

2. Copy number analysis consists of a series of accuracy estimates without even describing how accuracy was measured. From Fig 1b, it seems that the profile has a substantial level of noise, making CNV analysis difficult. Some basic QC analysis and a more careful CNV analysis is needed.

*Response: We agree with the reviewer’ opinion that CNV analysis required better data quality. As the CNV evaluation relied on reads counts mapped to the genome, we filtered single cells to remain the ones with genome coverage more than 10% to obtain more reliable results. We followed previous studies [5, 7, 8] to measure the accuracy of CNV results using the coefficient of variation (CV). As shown in Figure R3a [7] (Fu et al PNAS 2015), higher CV values means more noise and less accuracy, and the CV values decreased when we enlarge the bin size (Figure R3b) [8] (Chen et al Science 2017). Due to the relatively low read counts of Smile-seq on the genome, we analyzed CNVs using the K562 Smile-seq data with relatively large scales (1Mb in this study). Under this condition, the CV for all cells were quite reasonable and we could clearly identify the differential CNVs (on chromosome 7 and 9) between these two clones of K562 cells (Figure R3c). We added the explanations and citations in the revised manuscript.*

*Figure R3: CNV analysis. (a) This figure referenced from Fu et al PNAS 2015. (b) This figure referenced from Chen et al Science 2017. (c) Fig. 1b in the manuscript*

3. Because the cells were sequenced at such a low coverage, it is hard to interpret the accuracy estimates. For the purpose of benchmarking, higher coverage data would have been helpful to separate the impact of sequencing depth.

*Response: We appreciate the reviewer’s suggestion for obtaining higher coverage data, but at the moment the cost is too high to get much higher sequencing depth for an individual cell. We adopted Pacbio sequel II platform as generated highly accurate circular continuous consensus (CCS) reads by correcting multiple passes. Meanwhile, the total CCS reads of a flowcell were only about 15G-20G, corresponding to 16 single cells in our study. In this case, each single cell cost about $260. Yet the high cost of TGS sequencing prevented us from sequencing a single cell to much higher depth.*

*As displayed in Figure R4a, regardless of genome coverage (from 10% to ~40%), the precision of deletion detection for each cell is around 75%, and insertion 86%, indicating the high and stable performance of Smile-seq on deletion and insertion detection.*

*To make better benchmarking, we picked four K562 single cells with the highest genome coverage (35.7%-41.2%) to analyze the precision of our method. As shown in Figure R4b, more supported reads in the same cells could increase the accuracy of SV detection (we did not analyze duplications, translocations and inversions as there were quite limited number of calls in a single cell), and the false positive events largely reduced from 1 supported read to 2 supported reads in the same individual. In this way, further increasing the sequencing depth for a single cell would be helpful to further improve the accuracy of calling SVs if the high sequencing cost is not considered. We have added these new analyses into the main text following the reviewer’s suggestion.*

*Figure R4: (a) The genome coverage and the precision of insertion and deletion events detection (b) The precision and true positive numbers of detected insertion and deletion events under different supporting reads in four single K562 cells with high coverage.*

4. As an example, I will describe some of the issues with SNV/indel analysis. Although current estimates of PacBio CCS put the error rate at around 0.5% or less with proper error correction and consensus calling from multi-pass CCS data, the rate of erroneous indels and SNVs remains around 10-15% in raw data (Wenger et al 2019). Additionally, the length distribution of deletions (mostly <1-kb long) raises the concern that these deletions might represent sequencing errors. The authors do not present a clear analysis of how they reduce erroneous SNV and indel calls from long-read data. Specifically, the authors need to demonstrate that per single cell, they can identify and rule out SNV and <1kb-long indels that might originate from errors during sequencing. Four pieces of data from the authors suggest that many SNV, indel, and SV calls per single cell from their analysis might be errors.

*Response: Sorry that we did not make it clear in the previous version of our manuscript. Actually, we reduce false-positive SNV and indel calls from single cells of Smile-seq data using the following steps (as shown in the analysis workflow of Figure R5a):*

*1). We used CCS reads instead of raw subreads for analysis in Smile-seq. As shown in Figure R5b, over 95% CCS reads had over 5 passes, which corresponded to over 99% raw sequencing accuracy (Q20). Over 70% of the CCS reads had more than 10 passes, indicating 0.1% raw error rate (Figure R5c). Thus the reads were of high quality for SNV analysis.*

*Figure R5: A flowchart of reducing erroneous SNV and indel calls from long-read data (a) The analysis workflow of variations (including SVs, SNVs and indels) in K562 samples. (b)The distributions of the numbers of passes of CCS reads. (c) This figure referenced from Wenger et al nature biotechnology 2019. (d, e) The overlapping between insertions and deletions events detected by Smile-Seq and TGS bulk. (f) This figure referenced from Wenger et al nature biotechnology 2019. (g) The comparisons of precisions of deletions and insertions which less than 1kb and which over 1kb. (h) The precision and true positive numbers of detected insertion and deletion events under different supporting reads in four single K562 cells with high coverage.*

*2) We filtered SVs less than 100bp long. The TGS platforms were reported to have preference on false positive indels which usually less than 50bp for bulk samples [3, 9]. So in our analysis, we filtered SVs less than 100bp to avoid this problem. We believed that the SVs we identified were of high accuracy as majority of them overlapped with those found in TGS bulk sample, which is widely used as gold standard in the field (Figure R5d, e). Moreover, the previous study (Wenger et al 2019) has proved that the accuracy of SV detection using Pacbio sequencing platform did not change much for different lengths of SVs (longer than 50bp ones) (Figure R5f). To further check whether the short SVs (<1kb) have more false positive rates or not, we compared the precisions of SVs less than 1kb (100bp to 1kb) and over 1kb (Figure R5g). The precisions of both types of SVs increased with supported number of cells, while the precisions of <1kb SVs are comparable to those of >=1kb SVs, indicating that sequencing bias of Pacbio platform had minimal effects on calling SVs in our workflow.*

*3) We filtered SVs with only one supported read. As shown in Figure R5h, the accuracy of deletions called in single cells largely improved and the false positive SV calls reduced dramatically when asking two supported reads.*

4)	. To call SNVs in Smile-seq data, we ran GATK HaplotypeCaller v4.1.4.0. To reduce sequencing error for calling SNVs, we filtered low mapping quality and secondary aligned reads (the parameters for GATK is “--minimum-mapping-quality 60, --read-filter MappingQualityReadFilter, --read-filter NotSecondaryAlignmentReadFilter”). Moreover, the SNVs with QualByDepth < 2.0 (“--filter-expression QD < 2.0”) were removed. While even so, Smile-seq did not show good performance of calling SNVs in single cells as previous NGS based scWGS methods do. We think this is mainly because the base-substitution errors of PacBio platform is not random [4] and cannot be thoroughly removed by asking for two or more supported reads, even for bulk samples. Single cell analysis cannot improve this beyond the bulk samples. We have added the discussions in the revised manuscript.

a. First, in Figure 2c, the authors show how the percent of true-positive deletions drops off as more cells are required to share them. The near-linear decay in the percentages suggest that the number of deletions shared amongst a group of cells arises from random sampling (for example, a single cell has 71% of its deletions as true-positives, but the percent of deletions shared in two cells being positive suggests a product between the percentages of two independent cells, 0.716 * 0.716 = 0.516, which is close to the reported 55.2%). If the authors were capturing true clonal structure, then the decay in the percentage of shared deletions would deviate from the linear trend. The percentages do deviate from products of independent probabilities as more cells are required to support an event, but the trends for smaller groups of single cells suggest that many individual deletion calls per single cell are erroneous that require consensus calling to filter.

*Response: Sorry that we did not make it clear in the previous version of our manuscript. Actually, in the original Figure 2c (also see Figure R6a), the abscissa of this figure should be greater than or equal to (we have modified it accordingly), so the more cells we required, the less SV be supported. In fact, the average precision of deletion calls for single cell is 75.4%, and “71% on Figure 2c” refers to “>=1-cell supported true positive deletions/bulk TGS reference deletions”. The “near-linear decay” in the percentages (True positive calls/bulk TGS reference SVs) was mainly caused by the decreasing of genome coverage when we asked more supporting cells for the same genomic regions, and as a result, the number of multicell supported SV decrease. Here we first checked the downtrend of genome coverage in these two clones of K562 cells (Figure R6b), and then we counted the number of SVs supported by one to five, and over five single cells (Figure R6c). As illustrated by Figure R6c, if we require more supporting cells, the number of SV decreases, yet the precision of SV detection increases. Combining Figures R6a, R6b and R6c, we believe that the “near-linear decay percentage” can be clarified. In addition, we have analyzed the precision of calling each type of SVs in individual cells in Fig. 2b in the manuscript (also see Figure R6d). By filtered SVs with only one supported CCS read, the precision of SV calling in each single cell was about 75% (the duplication and translocation events were quite few), indicating that about 25% calls were erroneous. The false positive rate could be significantly reduced and the efficiency of detecting SVs could be largely increased when we include more individual cells in a group (Figure R6e).*

*Figure R6: (a) The precision of SV detection and the number of SV detected under different numbers of supporting cells. (b) The downtrend of genome coverage when asked more supporting cells. (c) The precision and true positive numbers of detected insertion and deletion events under different supporting cells in single K562 cells. (d) The precision of calling each type of SVs in individual cells in Fig. 2b in the manuscript. (e) The reduction of false positive rate and the increasing of SVs detecting efficiency when more individual cells are included in a group.*

b. Second, in the patient CRC sample, the authors find that 60% of SVs called from the CRC sample were shared in K562, and although the authors do focus on CRC-specific SVs, the high percentage suggests that the analysis of Smile-seq does not sufficiently filter out false-positive SV calls. The description of the PCR results in gDNA suggests that several SVs were found in all gDNA samples albeit at different sizes, suggesting false-positive mutation calls.

*Response: As aforementioned, we aligned our single-cell and bulk sequencing reads directly to the reference genome (hg38), and the SVs called in the third-generation sequencing (TGS) bulk samples were taken as gold standard to analyze the efficiency and accuracy of Smile-seq. The 60% of SVs called from the Smile-seq of CRC sample were shared in the K562 bulk TGS sample, so these shared SVs should not be false positive SV calls generated by Smile-seq analysis. Actually, since we align reads directly to the reference genome and call germline SVs, these SVs might be mainly inadequate human genome annotation (for SVs). In addition, we compared these two sets of SVs to those in HG002 reported by Zook et al. (Nature Biotechnology, 2020, ftp://ftp-trace.ncbi.nlm.nih.gov/giab/ftp/data/AshkenazimTrio/analysis/PacBio_pbsv_05212019/HG002_GRCh38.pbsv.vcf.gz) [1]. As shown in Figure R7, the SVs in each of these two sets shared quite a lot. Altogether, we believe that the high frequency of common SVs appeared in multiple datasets of different resources was caused by the inadequate human genome annotation (for SVs) and should not be due to drawbacks of Smile-seq method.*

*Figure R7: Overlapping between deletion events detected by Smile-seq, bulk TGS and reference HG002.*

c. Third, in the SNV analysis, it is not clear how the authors derived a false-positive rate of 2e-5, so clarification of how the authors estimated the false-positive rate is necessary. On the other hand, a back-of-the-envelope calculation based on the data suggests that the false discovery rate for SNVs per single cell might be higher than the authors estimate. Less than 20% of bulk-defined SNVs were captured in individual cells, suggesting that approximately 3,000 SNVs called per cell are either cell-specific or erroneous. This would roughly translate to about 17,000 total non-bulk SNVs per single-cell (if we assume that at most 17% of the cell's true SNVs are captured). These numbers would place the overall single-cell mutation frequency at approximately 35 mutations/Mb, which is seemingly high for K562 (and, based on MutSigCV analysis presented in Lawrence et al Nature 2013, this estimated rate would lie in the far-upper-tail of mutation rates for leukemias).Additionally, if the estimated precision per single cell for SNVs is approximately 10-20% (based on their cell-sharing analysis), then 80-90% of the mutation calls are likely to be false discoveries, which would suggest that most of the excess, non-bulk SNVs identified per single cell are likely false positives.

*Response: In previous version of our manuscript, we did not make it clear that we followed the previous published workflow [5] to calculate the SNV False positive rate (2e-5) in each single cell by dividing the number of the false positive SNVs by the total covered bases in a single cell. And this is comparable to previous scWGS methods (Figure R8a) [8]. This way we could make a comparison with the previous NGS platform-based scWGS methods. Due to relatively low sequencing depth in Smile-seq of K562 cells, we detected 16,488 SNVs on average in each single cell, in which only about 7% of NGS bulk-defined SNVs were captured in an individual cell. Finally, the overall single-cell mutation frequency was about 29mutations/Mb in K562 cells according to our analysis. This is a little higher than that presented in Zhou et al. Genome Research, 2019 [10] (23mutations/Mb).*

*We recognized the relatively poor performance of Smile-seq in SNV calling might be caused by the systemic error of TGS sequencing. While we usually do not use only one single cell to call SNV mutations in studies, the precision increases when we use more cells to correct the SNV results (Figure R8b).*

*In summary, as a scWGS method, Smile-seq performs comparable to some of previous methods in calling SNVs. We have added the discussions in the revised manuscript.*

*Figure R8: (a) This figure referenced from Chen et al., Science 2017. (b) The precision and numbers of true positive and false positive events for detected SNV under different number of supporting cells for single K562 cells*

d. Fourth, the authors report a consistent validation rate for insertions and SVs of ~50% (4 of 8 for K562 insertions, 1 translocation reported, and 5 of 8 insertions in CRC). Many of the events selected for experimental follow-up may not be prevalent enough across the clones to be well-validated, but the rate suggests that the authors' call set may contain many false or erroneous events.

*Response: Sorry that we did not make it clear in the previous version of our manuscript. As a complement, we also picked 8 insertion events in CRC to perform PCR validation, and 5 of 8 insertions had validated successfully. Since only 8 out of 1,376 insertion events were chosen, the ‘validation ratio’ (5/8) cannot be used to estimate the true positive or false positive rate (FPR). We think it is reasonable to use genome sequencing of bulk samples on the Pacbio platform as gold standard to estimate the true positive rate and false positive rate (FPR) and evaluate our smile-seq methods for SV detection.*

*The false positive rate (1-precision) should be less than 15% for calling deletion and insertion using Smile-seq data as we required the SVs to be supported by at least two cells (see Figure R9a, b). As mentioned in Question 4b, we deduce there were inadequate annotations in the current reference human genome, resulting in many common SVs across multiple data resources. And indeed, the unvalidated SVs in both K562 and CRC samples were appeared in all genomes we used for PCR validation, indicating that they were probably not false positive events in our analysis.*

*Figure R9: (a) The precision of calling each type of SVs in individual cells in Fig. 2b in the manuscript. (b) The Precision of Smile-seq detecting deletions and insertions the percentage of true positive deletions and insertions with different numbers of supporting K562 cells from merged clone 1 and clone 2.*

5. The authors should describe their quality control strategies in more detail or implement a workflow for distinguishing false-positive SNVs and indels per cell. The analysis of cell sharing is a good starting point, but the currently presented analysis does not sufficiently address how SNVs called from long reads even before cell-sharing analysis and at the step of read handling. The authors have not shown how these candidate mutation calls are separated into likely-false and likely-true calls, how the multiple passes or overlapping molecules are used to remove false SVs, and how <1kb-long deletions are sifted. The <1kb-long deletions are concerning because this represents a known error mode for long-read sequencing.

*Response: We appreciate the reviewer’s suggestions and added the workflow for calling and evaluating the SVs and SNVs in Smile-seq samples (Figure R10a). In SNV analysis, we used GATK to call SNVs in each single cell. After filtering by mapping quality, the SNVs were compared to those identified in bulk NGS data (~30x genome coverage). SNVs that were the same with those in bulk sample were regarded as true positive ones, and those different to bulk were defined as false positive calls. We used CCS reads (over 95% had at least 5 passes) for downstream analysis instead of using the raw subreads, thus to remove many false positive calls caused by sequencing errors (check Question4). As for SV analysis, we showed that the true positive ratio increased and the false positive ratio reduced dramatically when requiring at least 2 supporting reads (check Question4). In addition, we filtered indels less than 100bp long, which showed frequent sequencing errors in TGS platform. Besides, Wenger et al 2019 [3] has proved that the accuracy of SV detection using Pacbio sequencing was not affected by the lengths (when longer than 50bp) (Figure R10b). With further analysis on how the precision changes in SVs less than and longer than 1kb (Figure R10c), we believe the <1kb deletions here were not a major concern in calling SVs. We have added more details and discussions in the revised manuscript.*

*Figure R10: (a) The analysis workflow of variations (including SVs, SNVs and indels) in K562 samples. (b) This figure referenced from Wenger et al nature biotechnology 2019. (c) The comparisons of precisions of deletions and insertions which less than 1kb and which over 1kb.*

6. The authors do not establish how true SVs can be distinguished from false-positive SVs from low-coverage (average of 0.4x) single cell data. This problem is distinct from whether single cells can re-identify SVs from a bulk WGS sample of an ancestral or sibling clone. The latter problem is ultimately a measure of sensitivity, while the former problem is one of precision. The cell-sharing analysis provides an indirect sense of how many deletions might be false, but it is not clear how the authors filter out poor SVs from their initial call set before conducting cell-sharing analysis. Although the authors do use 10-pass CCS which should theoretically bring down the false-positive rate, the authors have not established whether filtering out erroneous bases is possible with sparse coverage. Furthermore, the lower coverage suggests that many events captured in one cell cannot be corroborated in another cell, since it is likely that not all loci will have at least one fragment for all cells. Ideally, the authors could sequence a portion of their cells at much higher overall coverage to show how many SVs are supported by separate long read fragments (and not just from multiple passes of the same CCS molecule).

*Response: Sorry we might not make it clear in the previous version of our manuscript that we only calculated the bases covered within each single cell. Following the standard accuracy evaluation process, we determined true and false positive SVs in each single cell by comparing to those called in the bulk TGS sample. We have discussed the precision of our Smile-seq data in our manuscript. We followed the reviewer’s suggestion to calculate the recall of SVs in Smile-seq data. To mitigate the impact of low sequencing coverage on single cells, we defined the recall as TP/(TP+FN) (herein, recall is equal to sensitivity by definition), where the FN represents the SVs not detected while the corresponding genome sites were covered in the single cell data. The averaged recall of deletions was 28.2% (insertions 19.3%) for a single K562 cell. We think this is mainly because K562 is near triploid (64.6% of K562 bulk TGS SV calls were heterozygous), and the coverage and depth of a single cell is not adequate to capture all SVs. However, by integrating single-cell SV calls and requiring multicell support, the recall of SV can improve (Figure R11a). For example, by requiring at least 2-cell supported SVs, the recall of deletions reached up to 73.9% (insertions 60.5%).*

*We have added the details of how we filter the false positive rate in single cells in the flowchart of Figure R11b. According to the answers to the first couple of questions, we believe that our workflow could largely reduce false positives in single cells. Moreover, it would be better to use a group of cells to further reduce false positive rate and increase the sensitivity.*

*As answered in Question#3, now it is difficult to sequence Smile-seq of a single cell to adequate depth due to the high sequencing cost. In our study of the K562 cells, each cell covered about 19% of the genome on average. There were in total of 91 K562 cells, and according to our dataset, over 95% of the genome was covered by at least 2 cells (Figure R11c). Therefore, sequencing a group of scWGS cells in relatively low depth is an alternative way than sequencing one cell very deeply to make more comprehensive analysis of the genome.*

*In bulk sequencing, it is easy to assemble different CCS reads to large fragments, and determine whether they contained SVs. While in scWGS, there are relatively high proportions of allele dropout during the single cell genome amplification process. Especially for transposon based single cell genome amplification, the CCS reads of contiguous transposed fragments of the same allele would not overlap with each other, made it difficult to assemble even when sequenced to very high depth. In this way, we could only determine SVs supported by CCS reads with scWGS data, such as Smile-seq.*

*Figure R11: (a) The Precision, Recall and F1 score of multicell-supported insertions and deletions. Precision= TP/(TP+FP), Recall=TP/(TP+FN), F1=2×Pre×Recall/(Pre+Recall). (b) The analysis workflow of variations (including SVs, SNVs and indels) in K562 samples. (c) The downtrend of genome coverage when asked more supporting cells.*

7. No discussions of inversions are provided. Past phased whole-genome analysis of K562 (Zhou et al Genome Biology 2019; Dixon et al Nature Genetics 2018) show the presence of many large inversions (63kb and 125kb inversions, for instance). Given that long reads have distinct advantages over short-read sequencing in resolving single or consecutive inversion events, we highly recommend an analysis of inversions from the K562 clones.

*Response: We followed the reviewer’s suggestions and added analysis on inversion events in the K562 cells (Figure R12). With long sequencing reads, Smile-seq detected more inversion events than the NGS bulk did [11], and the length could reach megabases, indicating advantages of Smile-seq in detecting inversion events than NGS based methods [10, 11]. We added these results in the revised manuscript.*

*Figure R12: The analysis of inversions events in K562 cells.*

8. The draft contains grammar and spelling errors that require careful proofreading.

*Response: Sorry for this and we have checked and corrected the grammar and spelling carefully in the revised manuscript.*

*References*

*1.	Zook JM, Hansen NF, Olson ND, Chapman L, Mullikin JC, Xiao C, Sherry S, Koren S, Phillippy AM, Boutros PC, et al: A robust benchmark for detection of germline large deletions and insertions. Nat Biotechnol 2020, 38:1347-1355.*

*2.	Sedlazeck FJ, Rescheneder P, Smolka M, Fang H, Nattestad M, von Haeseler A, Schatz MC: Accurate detection of complex structural variations using single-molecule sequencing. Nature Methods 2018, 15:461-468.*

*3.	Wenger AM, Peluso P, Rowell WJ, Chang PC, Hall RJ, Concepcion GT, Ebler J, Fungtammasan A, Kolesnikov A, Olson ND, et al: Accurate circular consensus long-read sequencing improves variant detection and assembly of a human genome. Nat Biotechnol 2019, 37:1155-1162.*

*4.	Dohm JC, Peters P, Stralis-Pavese N, Himmelbauer H: Benchmarking of long-read correction methods. NAR Genom Bioinform 2020, 2:lqaa037.*

*5.	Zong C, Lu S, Chapman AR, Xie XS: Genome-wide detection of single-nucleotide and copy-number variations of a single human cell. Science 2012, 338:1622-1626.*

*6.	Huang L, Ma F, Chapman A, Lu S, Xie XS: Single-Cell Whole-Genome Amplification and Sequencing: Methodology and Applications. Annual Review of Genomics and Human Genetics 2015, 16:79-102.*

*7.	Fu Y, Li C, Lu S, Zhou W, Tang F, Xie XS, Huang Y: Uniform and accurate single-cell sequencing based on emulsion whole-genome amplification. Proc Natl Acad Sci U S A 2015, 112:11923-11928.*

*8.	Chen C, Xing D, Tan L, Li H, Zhou G, Huang L, Xie XS: Single-cell whole-genome analyses by Linear Amplification via Transposon Insertion (LIANTI). Science 2017, 356:189-194.*

*9.	Logsdon GA, Vollger MR, Eichler EE: Long-read human genome sequencing and its applications. Nat Rev Genet 2020, 21:597-614.*

*10.	Zhou B, Ho SS, Greer SU, Zhu X, Bell JM, Arthur JG, Spies N, Zhang X, Byeon S, Pattni R, et al: Comprehensive, integrated, and phased whole-genome analysis of the primary ENCODE cell line K562. Genome Res 2019, 29:472-484.*

*11.	Dixon JR, Xu J, Dileep V, Zhan Y, Song F, Le VT, Yardımcı GG, Chakraborty A, Bann DV, Wang Y, et al: Integrative detection and analysis of structural variation in cancer genomes. Nature Genetics 2018, 50:1388-1398.*

**Second round of review**

**Reviewer 2**

I have asked a number of questions in my first review. Unfortunately, I do not feel that they have addressed them. Their response address some technical aspects, but they do not seem to have addressed the bigger questions. The response is repetitious and the changes to the main text/figures are minimal. The overall idea of Smile-seq (PacBio+low concentration of transposase) is fine and that you can increase accuracy by taking variants that are shared in two (or more) cells is reasonable. More plots with various precision/recall type information are provided in the revised version, but I think the authors are still far from demonstrating that their method can be used to identify accurate somatic mutations in single cells.

Rather than going through each section of the long response, I will just mention two of the key concerns here.

The question of germline vs somatic variants is an important one. The abstract mentions analysis of "human cancer cell lines and a colorectal cancer sample," which most readers will interpret to mean that you are analyzing somatic variants. After all, people do not do single cell sequencing to identify germline variants. The authors have in their response clarified that they are in fact doing germline analysis. Given this information, I would argue that most of the estimates for false positives, false negatives, etc. are invalid for somatic analysis and the analysis needs to be redone. When analysis is done for germline variants in single cells without subtracting the germline variants from the bulk sample, the denominator (the number of SNPs) in the analysis is very large, and the estimates are dramatically skewed. Thus estimates in the paper are totally misleading for any somatic analysis that people may do.

As an another example, I asked for more systematic analysis for CNV, but the authors only discuss coefficient of variation (CV). CV is a measure of data quality and is related to CNV analysis, but it is not CNV analysis. In the main text, basically the only CNV analysis is that they see the two clones of K562 having differences in the two chromosome arms. I think this is very far away from "reliably and effectively detecting CNVs" as the abstract states.

**Authors Response**

**Point-by-point responses to the reviewers’ comments:**

Reviewer #2: I have asked a number of questions in my first review. Unfortunately, I do not feel that they have addressed them. Their responses address some technical aspects, but they do not seem to have addressed the bigger questions. The response is repetitious and the changes to the main text/figures are minimal. The overall idea of Smile-seq (PacBio+low concentration of transposase) is fine and that you can increase accuracy by taking variants that are shared in two (or more) cells is reasonable. More plots with various precision/recall type information are provided in the revised version, but I think the authors are still far from demonstrating that their method can be used to identify accurate somatic mutations in single cells.

Rather than going through each section of the long response, I will just mention two of the key concerns here.

The question of germline vs somatic variants is an important one. The abstract mentions analysis of "human cancer cell lines and a colorectal cancer sample," which most readers will interpret to mean that you are analyzing somatic variants. After all, people do not do single cell sequencing to identify germline variants. The authors have in their response clarified that they are in fact doing germline analysis. Given this information, I would argue that most of the estimates for false positives, false negatives, etc. are invalid for somatic analysis and the analysis needs to be redone. When analysis is done for germline variants in single cells without subtracting the germline variants from the bulk sample, the denominator (the number of SNPs) in the analysis is very large, and the estimates are dramatically skewed. Thus estimates in the paper are totally misleading for any somatic analysis that people may do.

*Response: In the past ten years, several milestone NGS platform-based single-cell genome amplification techniques have been developed, such as DOP-PCR (Navin et al Nature, 2011(only analyzed CNVs)), multiple displacement amplification (MDA) (Wang et al Cell, 2012), multiple annealing and looping-based amplification cycles (MALBAC) (Zong et al Science, 2012), Linear Amplification via Transposon Insertion (LIANTI) (Chen et al Science, 2017), and Multiplexed end-tagging amplification of complementary strands (META-CS) (Xing et al PNAS, 2021) (as shown in the following Figure R1-R5). While in these methods, all of them evaluated the accuracy of calling SNVs in scWGS data by analyzing the cell line-specific SNVs happened in every individual cell of the cultured cancer cell lines or normal cell lines or in in vivo cells (i.e. the human sperms). From the frequency point of view, they are equivalent to the germline point mutations that happened in 100% of the cells analyzed, but not subclonal point mutations that happened in 20% of the cells analyzed. And none of them ever evaluated the accuracy of calling SNVs in scWGS data by analyzing the subclone-specific SNVs (that happened in 20% of the cells analyzed). Following the same logic, we calculated the accuracy of our Smile-seq method in detecting genomic variants (both SNVs and structural variants). Taking advantage of long sequencing reads from the third-generation sequencing platform, to our best knowledge, our Smile-seq is noteworthy the first method aiming to profile the genomic structure variations at single-cell level. According to our thorough evaluations, Smile-seq has showed acceptable, if not ideal, performance on structural variation detection in K562 cancer cell line (with precision over 0.8 for SVs).*

*In the previous comments of Reviewer #2, the reviewer emphasized the importance of analyzing germline variants (and we quote ‘germline variants should be studied in great detail first, so that you can take advantage of a gold standard set of variants’), insisting that it should be the gold standard and basic task. We are delightful to accept Reviewer 2’s suggestion, and as we have explained in our response, we actually have investigated the performance of Smile-seq on ‘germline’ variant detection (From the frequency point of view, they are equivalent to the germline variants, that happened in 100% of the cells analyzed, but not subclonal variants that happened in 20% of the cells analyzed). During this analysis, we have excluded all of the known SNP sites (real germline variations) from both the bulk NGS sequencing samples and single cell Smile-seq samples.*

*For the point mutation detection, Reviewer #2 seems not read our description carefully. When we analyzed the SNVs in K562 cancer cell line, we excluded all of the known SNP sites from both the bulk NGS sequencing samples and single cell Smile-seq samples (This is a common sense in the field) (in the main text, page #10 line #234: ‘and to remove the impact of single nucleotide polymorphism (SNP) sites, we removed SNP sites when searching the candidate mutations.’) So with respect to the K562 cell line, the SNVs identified from the bulk NGS sequencing samples were ‘germline’ mutations (because from the frequency point of view, they happened in 100% of the cells analyzed). Meanwhile, these SNVs were also K562 specific somatic point mutations (From the point of view that they do not happen in normal human tissues in vivo. That’s why only～70,000 SNVs identified in the K562 bulk sample, instead of one million ‘SNVs’ identified). And this is the same for all the SNVs identified from the single cell Smile-seq samples. We know that there are two different types of the real somatic mutations in tumor samples in vivo: the point mutations happened in every cancer cell in the tumor (in 100% of the cells analyzed) and the point mutations happened in subclones of cancer cells in the tumor (in 20% of the cells analyzed).*

*We understand Reviewer 2’s concern on somatic variant detection. Theoretically, with tumor and matched normal samples, people can detect somatic variants by subtracting germline variants in normal controls from those in corresponding tumor samples. Since we have demonstrated the efficiency and accuracy of Smile-seq on ‘germline’ structural variant detection, it is reasonable to argue the capability of Smile-seq on detecting somatic structural variants. However, even for TGS-based genome sequencing of bulk samples using millions of cells, currently no golden standard is available for calling somatic structural variants for a real human tumor sample [1, 2]. Therefore, no matter in previous studies (single cell NGS-based genome sequencing methods) or this study, we all did not evaluate the performance of the method in calling subclone specific somatic variants in real tumor samples. Anyway, we do apply Smile-seq to test an in vivo sample from a patient with colorectal cancer. We have showed that the SVs called by Smile-seq data in cancer samples could be validated properly (although we only chose just several SV events to verify), demonstrating the power of Smile-seq in SV detection.*

As an another example, I asked for more systematic analysis for CNV, but the authors only discuss coefficient of variation (CV). CV is a measure of data quality and is related to CNV analysis, but it is not CNV analysis. In the main text, basically the only CNV analysis is that they see the two clones of K562 having differences in the two chromosome arms. I think this is very far away from "reliably and effectively detecting CNVs" as the abstract states.

Response: As for CNV analysis, our target is to illustrate that Smile-seq can be used to infer the CNVs in the genome with a reasonable bin size of about 1Mb. We have calculated coefficient of variation (CV) to measure the noise of calling CNVs with Smile-seq data. As we have illustrated in the manuscript, Smile-seq achieved acceptable CV values at single cell level and could discriminate the differences of genomic copy numbers between the two K562 clones. We have shown two aspects in the previous version of our manuscript: (1) the CNV patterns we called from a single cell is very similar to the CNV patterns from NGS-based bulk samples (2) The CV of the CNV patterns in a single cell (when compared with the average levels of CNV patterns from all of the single cells analyzed) is reasonably low.

*It seems that Reviewer #2 did not get this point. We now showed this in much more straightforward way. In our previous version, the baseline of copy number used to calculate CV is the mean value of K562 single cells (the average CV value is 0.28 for all of the single cells analyzed). In order to verify our results, we recalculated the CV using bulk K562 cells as the baseline, and the results are not much different from the previous ones. If we called the CNVs systematically wrongly in our single cell dataset, when we use bulk K562 cells as the baseline instead of the mean value of K562 single cells as the baseline, the CV will become much worth (much higher). But what we see is that the CV is till reasonable low (the average CV value is 0.29 for all of the single cells analyzed). Furthermore, we plotted the mean CNV values of K562 single cells to the CNV values of K562 bulk samples. And you can clearly see that they are highly consistent (as shown in the following Figure R6), demonstrating that the CNV results we calculated using Smile-seq data were reliable. We have added additional analysis and results into main text (Figure 1b).*

*References*

*1.	Lee AY, Ewing AD, Ellrott K, Hu Y, Houlahan KE, Bare JC, Espiritu SMG, Huang V, Dang K, Chong Z, et al: Combining accurate tumor genome simulation with crowdsourcing to benchmark somatic structural variant detection. Genome Biol 2018, 19:188.*

*2.	Cameron DL, Di Stefano L, Papenfuss AT: Comprehensive evaluation and characterisation of short read general-purpose structural variant calling software. Nat Commun 2019, 10:3240.*

**Third round of review**

**Reviewer 2**

There is almost no changes to the manuscript text. Instead, in their response, the authors claim that their analysis approach follows what other prominent papers have done in the past. The authors state that “Reviewer #2 seems not read our description carefully”. I in fact read the relevant sections multiple times but could not figure out exactly what was being done. I would think that I am an above-average reader in terms of my expertise n the topic. Either the text is so unclear that I remain confused, or I would encourage the authors to read those papers they cite and re-examine their argument.

The most recent paper they cite is META-CS from Sunney Xie’s lab (Xing et al, PNAS 2021). There, they perform clonal expansion of a single ancestor from a cell line, and they identify de novo SNVs in single cells compared to the ancestor cell. (To me, this is equivalent to the analysis of “subclonal” mutations that the authors claim is never done) These de novo mutations are generally <100 per cell. Their estimated false positive rate is 2.4*10^-8.

In the submitted paper, they also expand from single ancestors from a cell line and then sequence bulk and single cells. But then they remove only the polymorphic variants (known SNPs), before comparing the results. The authors have an average of 16,000 SNVs in a single cell (with only ~7% genome coverage) and estimated the false positive rate is 2.0 x 10^-5. It seems to me that what the authors did here is different from what Xie et al have done. It is also different from many other papers on single cell analysis that have done, e.g., kindred cell analysis. Some analysis, e.g., sensitivity measurements, can be performed using all SNPs. But other measures cannot be obtained accurately when the sample-specific variants are not properly considered.

Also, at this false positive rate the authors state in their analysis (if it is correct), I’m not sure what problem would be appropriate. That FPR surpasses the observed somatic mutation rates of nearly all tumor types I know (melanomas and mismatch repair-deficient tumors may be around >20 SNVs per mega base). I don't know whether the authors are aware of this and choose to not mention it.

The authors may argue, correctly, that the advantage of the long-read data is CNV/SV analysis rather than SNVs. But the CNV part remains inadequate—I don’t know of (good) papers where the coefficient of variation and visual inspection are used as a metric when claiming a new protocol/method.

**Reviewer 3**

The authors did not assess the ability of their method to detect somatic mutations (only germline) in single cells. In the current results section (lines 231-246) this is not stated clearly, the authors imply that they have removed SNPs and are not only evaluating somatic mutations (which they are not, since they cannot do this accurately without a matched normal tissue or cell line). As such their FP error rate is likely based on many germline SNVs and is likely much higher for somatic mutations. I do not agree that this additional data needs to be added to the currently manuscript, but that it needs to be stated very clearly so that readers are not misled, that the manuscript did not asses any error rates or ability to detect somatic mutations.

The argument that it’s not possible to run human tumor samples, of course is not valid. Also, there are many cell lines of paired normal and cancer cells that could be used to establish FP error rates for somatic mutation calls.

The overall analysis of copy number data in this study is poor at best, which is problematic since its one of the major focuses of the paper (in addition to SV analysis). The only metric they calculate is CV which is a measure of noise. Copy number data in Figure 1b is impossible to assess in terms of data quality and reproducibility, since it requires a visual comparison of the plots which is not acceptable. The data should be segmented with a segmentation algorithm (eg. CBS) and the resulting data should be shown as a heatmap.

The authors should also compute a pearson correlation of the single cells to the Bulk K562 gold standard to have a quantitative measure of how correlated the data is. In the current form, its impossible to asses the reproducibility and quality of the CNA data. Also 1mb resolution is quite high, most single cell studies these days have been able to measure genomic copy number at 50-200kb resolutions. The investigators should demonstrate that data quality at different resolutions eg. 1mb, 500kb, 200kb, 100kb to asses its usefulness for detecting copy number aberrations. A more rigorous and quantitative analysis is needed for this section of the paper.

The FP error rates are not being estimated accurately and include most germline variants (SNPs) in their calculations, not somatic mutations that can occur at different frequencies than homozygous/heterozygous (1.0 or 0.5) allele frequencies. The CNV analysis remains poor and based mainly on CV and ‘visual inspection’ of plots. My suggestion is to deemphasize (or even remove?) the mutation analysis and refocus the paper on the SV detection, which is the strongest part of the paper and the main reason for doing long-read sequencing analysis. The claim in the final conclusions that ‘This method reliably and effectively detected CNVs, SVs and ecDNAs in individual cells’ is currently not supported for CNVs with any quantitative metrics. Finally, the writing of the paper could be greatly improved for grammar and scientific language.

**Authors Response**

**Point-by-point responses to the reviewers’ comments:**

Additional reviewer (Reviewer #3): The authors did not assess the ability of their method to detect somatic mutations (only germline) in single cells. In the current results section (lines 231-246) this is not stated clearly, the authors imply that they have removed SNPs and are not only evaluating somatic mutations (which they are not, since they cannot do this accurately without a matched normal tissue or cell line). As such their FP error rate is likely based on many germline SNVs and is likely much higher for somatic mutations. I do not agree that this additional data needs to be added to the currently manuscript, but that it needs to be stated very clearly so that readers are not misled, that the manuscript did not asses any error rates or ability to detect somatic mutations.

The argument that it’s not possible to run human tumor samples, of course is not valid. Also, there are many cell lines of paired normal and cancer cells that could be used to establish FP error rates for somatic mutation calls.

*Response: Thanks very much to Reviewer 3’s comments. We have added “It is worth mentioning that we were detecting ‘germline-like’ mutations of the K562 cells, since it is a cultured cell line which was subcloned already” in the revised manuscript (Line 241-242).*

Additional Reviewer (Reviewer #3): The overall analysis of copy number data in this study is poor at best, which is problematic since its one of the major focuses of the paper (in addition to SV analysis). The only metric they calculate is CV which is a measure of noise. Copy number data in Figure 1b is impossible to assess in terms of data quality and reproducibility, since it requires a visual comparison of the plots which is not acceptable. The data should be segmented with a segmentation algorithm (eg. CBS) and the resulting data should be shown as a heatmap.

The authors should also compute a pearson correlation of the single cells to the Bulk K562 gold standard to have a quantitative measure of how correlated the data is. In the current form, its impossible to asses the reproducibility and quality of the CNA data. Also 1mb resolution is quite high, most single cell studies these days have been able to measure genomic copy number at 50-200kb resolutions. The investigators should demonstrate that data quality at different resolutions eg. 1mb, 500kb, 200kb, 100kb to asses its usefulness for detecting copy number aberrations. A more rigorous and quantitative analysis is needed for this section of the paper.

*Response: We do appreciate the reviewer’s suggestions and added circular binary segmentation (CBS) algorithm to segment DNA copy number and plot the heatmap (Fig. R1a). As illustrated by Fig. 1b, (also see Fig. R1b), we can still observe distinct CNV patterns between these two clones of K562 cells in chromosomes 7 and 9. We have added the heatmap in the revised Fig. S4.*

*Fig. R1: (a) The heatmap of K562 copy number. (b) CNVs of single K562 cells showing in 1Mb windows.*

*Besides, we also computed Pearson correlations coefficients of segmented copy numbers between any two individual cells as well as between the single cells and the bulk samples (Fig. R2a and Fig. R2b, all P-values < 1×10-5). As displayed by Fig. R2a and R2b, the Pearson correlation of copy number between bulk K562 samples and single cells were around 0.75, except for one cell showing relatively low correlation around 0.5. This indicates well reproducibility for calling CNVs using Smile-seq. data.*

*Fig. R2: The heatmap and distribution of the Pearson correlation coefficients of CNV calling (P-values < 1×10-5)*

*We understand the reviewer’s concern on the resolution of bin sizes. Actually, we have tried smaller bin sizes such as 100kb and 10kb, where the performance of Smile-seq was poor. We inferred that this was caused by the far less number of reads in Smile-seq data than those in the NGS data when acquired with the same genome coverage. Actually, the average read length of Smile-seq is about 6kb, which is about 20 times of NGS read length (150bp×2, pair-end). Therefore, with the same amount of data (bases), the number of mapped reads of Smile-seq was about only 1/20 of that from NGS platform. As a result, the reads coverage for each bin in calculating the CNV is far less in Smile-seq single cell data of 19% genome base coverage, which caused more bias in the copy number estimation for smaller bin sizes. As the reviewer mentioned, nowadays single-cell NGS studies can measure genomic copy number at 50-200kb resolutions. If we multiple the resolutions with 20, a reasonable resolution of Smile-seq would be at 1-4Mb, and the 1Mb resolution we claimed for Smile-seq in this study just drops in this interval.*

*Thanks again for Reviewer 3’s helpful comments, we have quantitatively measured Smile-seq CNV analysis. Overall, Smile-seq has shown acceptable performance on CNV analysis, although the resolution is relatively low. We have revised the statement as “Smile-seq reliably and effectively detected SVs and ecDNAs in individual cells, but showed relatively limited accuracy in detection of CNVs and SNVs” in the revised manuscript (Line 22-23).*

Additional Reviewer (Reviewer #3): The FP error rates are not being estimated accurately and include most germline variants (SNPs) in their calculations, not somatic mutations that can occur at different frequencies than homozygous/heterozygous (1.0 or 0.5) allele frequencies. The CNV analysis remains poor and based mainly on CV and ‘visual inspection’ of plots. My suggestion is to deemphasize (or even remove?) the mutation analysis and refocus the paper on the SV detection, which is the strongest part of the paper and the main reason for doing long-read sequencing analysis. The claim in the final conclusions that ‘This method reliably and effectively detected CNVs, SVs and ecDNAs in individual cells’ is currently not supported for CNVs with any quantitative metrics. Finally, the writing of the paper could be greatly improved for grammar and scientific language.

*Response: We really appreciate the reviewer’s suggestions and revised the statement in the manuscript to avoid misleading. Since Smile-seq is, to our best knowledge, the first single-cell WGS method based on third-sequencing technologies, we believe that it is necessary to report its competences, including both advantages and drawbacks in the first version. Therefore, we would like to keep the results of SNVs and CNVs analysis but tone down them.*
